# Genomic evolution and adaptation of arthropod-associated *Rickettsia*

**DOI:** 10.1038/s41598-022-07725-z

**Published:** 2022-03-09

**Authors:** Khalid El Karkouri, Eric Ghigo, Didier Raoult, Pierre-Edouard Fournier

**Affiliations:** 1 Aix-Marseille University, IRD, AP-HM, SSA, VITROME, Marseille, France; 2 IHU Méditerranée Infection, Marseille, France; 3 Aix-Marseille University, IRD, AP-HM, MEPHI, Marseille, France

**Keywords:** Evolution, Microbiology, Diseases, Pathogenesis

## Abstract

*Rickettsia* species are endosymbionts hosted by arthropods and are known to cause mild to fatal diseases in humans. Here, we analyse the evolution and diversity of 34 *Rickettsia* species using a pangenomic meta-analysis (80 genomes/41 plasmids). Phylogenomic trees showed that *Rickettsia* spp. diverged into two Spotted Fever groups, a Typhus group, a Canadensis group and a Bellii group, and may have inherited their plasmids from an ancestral plasmid that persisted in some strains or may have been lost by others. The results suggested that the ancestors of *Rickettsia* spp. might have infected Acari and/or Insecta and probably diverged by persisting inside and/or switching hosts. Pangenomic analysis revealed that the *Rickettsia* genus evolved through a strong interplay between genome degradation/reduction and/or expansion leading to possible distinct adaptive trajectories. The genus mainly shared evolutionary relationships with α-proteobacteria, and also with γ/β/δ-proteobacteria, cytophagia, actinobacteria, cyanobacteria, chlamydiia and viruses, suggesting lateral exchanges of several critical genes. These evolutionary processes have probably been orchestrated by an abundance of mobile genetic elements, especially in the Spotted Fever and Bellii groups. In this study, we provided a global evolutionary genomic view of the intracellular *Rickettsia* that may help our understanding of their diversity, adaptation and fitness.

## Introduction

*Rickettsia* species (spp.) (Order Rickettsiales, Family *Rickettsiaceae*) are obligate intracellular α-proteobacteria of both ecological and medical interest. These bacteria live in close association with a diverse range of hosts including arthropods, mostly, and vertebrates, plants, algae, annelids, amoebae, ciliate and medusae (as primary hosts and vectors)^1–6^. *Rickettsia* spp. are transmitted to humans or animals, mostly through arthropod bites, and are responsible for mild to fatal diseases such as epidemic typhus and Rocky Mountain spotted fever^3,7^.

Obligate intracellular bacterial endosymbionts and pathogens evolve in diverse host environments. These biological and ecological lifestyles make their genetic manipulations and understanding the mechanisms of their survival and pathogenicity^8,9^ difficult. To overcome this barrier, evolutionary biology using comparative genomics is a powerful tool to decipher genomic signatures that have shaped bacterial genomes and to understand their diversification, adaptation and fitness. Earlier investigations of *Rickettsia* spp. using comparative genomics have revealed several genetic and evolutionary features within these small (1.1–2.3 Mbp) genomic bacteria, such as (i) a high degree of inter-species genomic synteny; (ii) reductive evolution possibly in relation to their strict intracellular lifestyle; (iii) an enrichment in A + T content, polyA/T homopolymers; (iv) a lack of some metabolic pathways for which host cells provide the missing metabolites; (v) conjugative machinery and mechanisms of adhesion to host and motility; and (vi) a variable distribution of plasmids^10–28^.

Although initially thought to be devoid of plasmids, several *Rickettsia* spp. were demonstrated to harbour such mobile genetic elements (MGEs)^27^. While no obvious association between pathogenicity and acquisition of virulence factors was found in *Rickettsia* spp.^20^*,* other studies associated the increased virulence with genome reduction in this genus^29^. However, due to the absence of several genome sequences of the genus *Rickettsia* in public databases, all these comparative genomic investigations were carried out from subsets of species, sometimes drawing conclusions for a species by using a single strain.

In this study, we investigated the genomic evolution and diversity of the obligate intracellular *Rickettsia* spp. using phylogenomic and pangenomic meta-analyses from 34 arthropod-associated species which are represented by 80 genome sequences that we here reannotated. We investigated both the chromosomes and neglected plasmids, as both replicons are part of the historical life of this genus.

## Results

In this study, we examined the diversity, evolution and adaptation of the genus *Rickettsia* from 34 species with validated names. The 80 studied strains had been isolated from diverse hosts between 1938 and 2012 worldwide (Table S1). In order to obtain a robust comparative genome analysis, we performed standard re-annotations of 61 complete genomes and 19 draft genomes sequences. Their general genomic features are summarised in Supplementary Table S1.

### Phylogenomics and diversity

**Figure 1:**
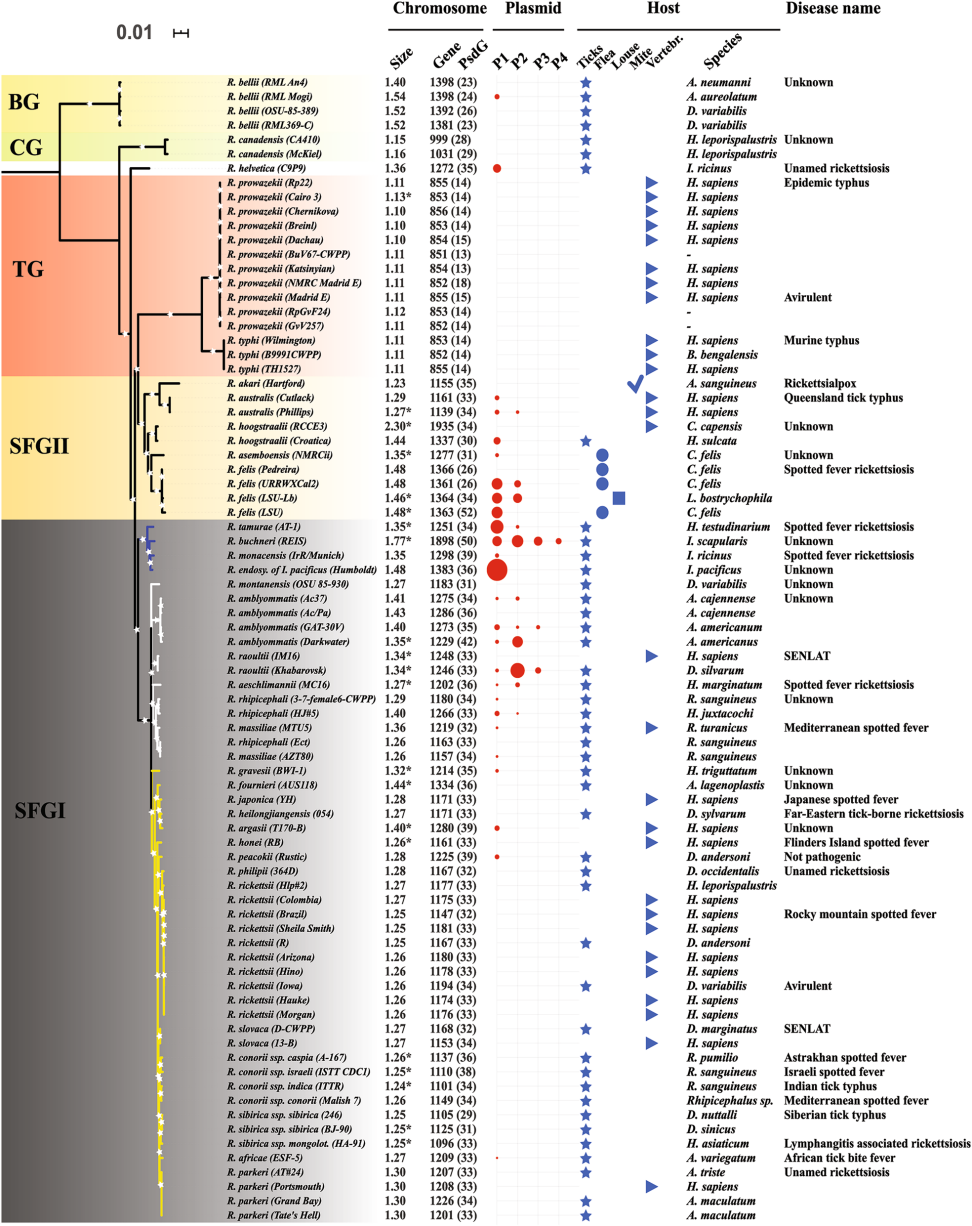
Maximum likelihood phylogenomic tree of arthropod-associated *Rickettsia* spp. estimated from 461 core chromosomal genes containing 36,880 core genes of 34 species and 80 strains. For each strain, the columns show its chromosome size, gene content (pseudogenes in %), number of plasmids and their sizes (presented by the diameter of circles) and host (either familiar or taxonomic name), as well as the name of the disease that it causes in humans. *A*: *Amblyomma*, *H*: *Homo sapiens*, *D*: *Dermacentor*, *R*: *Rhipicephalus*, *A. lagenoplastis*: *Argas lagenoplastis*, *H*. *leporispalustris/juxtacochi/sulcata*: *Haemaphysalis leporispalustris/juxtacochi/sulcata*, *H. asiaticum/marginatum/testudinarium/triguttatum*: *Hyalomma asiaticum/marginatum/testudinarium/triguttatum*, *I*: *Ixodes*, *A*. *sanguineus*: *Allodermanyssus sanguineus*, *C*: *Ctenocephalides*, *L*: *Liposcelis*, *C*. *capensis*: *Corvus capensis* and *B*: *Bandicota*.

We first inferred a Maximum Likelihood phylogenomic tree from 461 concatenated core chromosomal proteins of the 80 studied strains (Fig. 1). Overall, our robust evolutionary tree showed that the *Rickettsia* genus has diverged into five distinct and well-supported major groups (BP = 100%): the Spotted Fever groups (SFGI and SFGII), the Typhus group (TG), the Canadensis group (CG) and the Bellii group (BG).

The SFGI rickettsiae group included several closely related species among which the core genome is highly conserved as reflected by short branches in the tree. This lineage is clearly split into two distinct sister sub-clades of rickettsiae (BP node supports = 100%), SFGIa and SFGIb, containing 20 and four species (*e.g., R. conorii* and *R.* endosymbiont of *I. scapularis*), respectively. The next well-resolved group is designated here as the SFGII rickettsiae group (also referred to as the transitional group, TRG), which includes five spotted fever rickettsial species (*e.g., R. felis* and *R. australis*). The core genome of this group appears to be more divergent compared with the SFGI group.

Another member of the SFG species, *R. helvetica*, was placed as an individual clade basal to the TG group but not the SFG group (Fig. 1). Examination of the multiple sequence alignments of *Rickettsia* core genes revealed that *R. helvetica* shares several non-synonymous mutations and large insertions/deletions with the SFGII, TG, CG and/or BG rickettsiae (Supplementary Fig. S1).

Remarkably, the TG group is clearly monophyletic and displays the longest and most diversified branch in the tree as compared with its close relatives (Fig. 1). This group accumulated several mutations in its core genome and then recently diverged into the *R. prowazekii* and *R. typhi* species. Last, the CG and BG groups were placed on two external branches of our phylogenomic tree. The former group includes *R. canadensis* and is clearly basal to the SFG and TG groups, whereas the latter contains *R. bellii* and is the earliest divergent outgroup among studied taxa.

### Plasmids

Our genomic annotation detected 41 rickettsial plasmids, of which 11 were newly identified (Fig. 1, Table S1). These plasmids are present in 56% and 33% of examined species and strains, respectively. Thus, 44% and 67% of species and strains, respectively, had no plasmids. By mapping the current large-scale plasmid data set over the five rickettsial phylogroups (SFGI, SFGII, TG, CG and BG), we found that rickettsial plasmids were variably distributed between and within these lineages. They were present in only three phylogroups (SFGI, SFGII and BG), with between one and four plasmid(s) per strain. A large number of rickettsial plasmids (three or four) were only observed in three SFG species, *R. raoultii*, *R. amblyommatis* and *R. buchneri*.

Interestingly, we detected typical plasmidic genes in the chromosomes from the 11 plasmidless *R. prowazekii* strains and the two *R. raoultii* strains (one exhibiting three plasmids and the other being plasmidless). Indeed, each of the plasmidless *R. prowazekii* strains showed a cluster containing five pseudogenes (RprME0791 to RprME0807) that exhibited high homologies (ids = 67–92%) with rickettsial plasmid genes. Similarly, both *R. raoultii* strains harboured a cluster of six genes (Rra_909 to Rra_0916) that best matched (ids = 82–97%) six genes present exclusively in the *R. peacockii* plasmid (pRpe).

**Figure 2:**
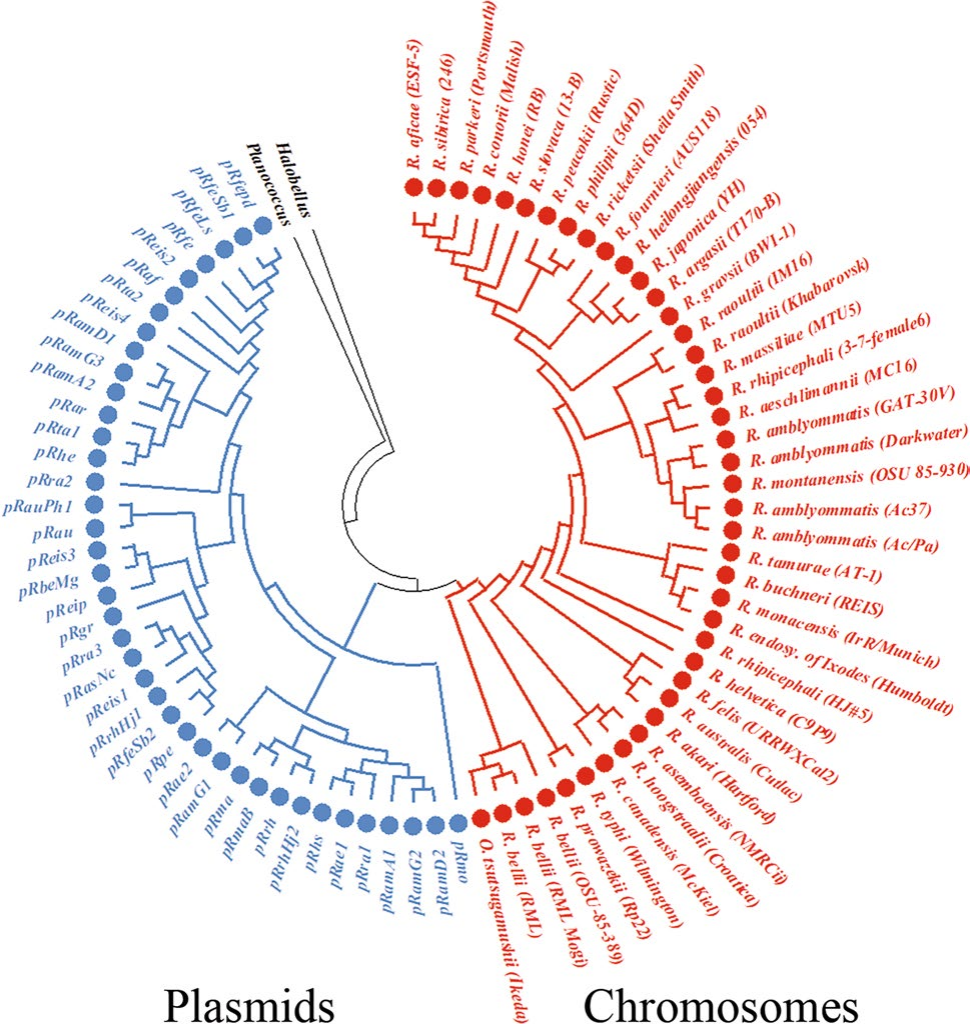
Supertree obtained from five genes of *Rickettsia* plasmids (n = 40) and chromosomes (n = 42) using the Robinson-Foulds algorithm (RF distance = 181). *Orientia tsutsugamushi* Ikeda (NC_010793.1), *Planococcus antarcticus* (ZP_10207867.1) and *Halobellus rufus* (WP_049986146.1) were used as best homologues to rickettsial genes. The list of genes used in the analysis includes dnaA-like replication initiator protein, helix-turn-helix DNA-binding domain, patatin-like phospholipase, cell surface antigen Sca12 and small heat shock protein (Supplementary Fig. S2).

The phylogenetic trees computed from 5 selected genes common to several plasmids (Supplementary Fig. S2) and identified using the pan-plasmidome analysis revealed that each displayed two major ancestral nodes, one gathering all plasmids and the other all chromosomes of *Rickettsia/Orientia*. These results clearly show that these genes originated from a *Rickettsia/Orientia* ancestor harbouring one ancestral chromosome and one ancestral plasmid. Moreover, the supertree constructed from these five individual gene trees confirmed the distinction of both phylogenetic clades and their nodes, which were obtained in each of the 5 phylogenetic trees (Fig. 2).

### Arthropod hosts

We compared the diversity of arthropods (as the primary hosts) associated with *Rickettsia* spp. across the five rickettsial phylogroups (Fig. 1). We found that most SFGI *Rickettsia* spp. were mainly associated with ticks (Acari), similarly to CG and BG rickettsiae. For example, the SFGI group was associated with seven genera and 27 species of ticks (*Amblyomma*, *Argas*, *Dermacentor*, *Haemaphysalis*, *Hyalomma*, *Ixodes* and *Rhipicephalus*). Moreover, some SFGI, CG and BG *Rickettsia* spp. were found to be associated with the same tick genus and/or species. However, SFGII rickettsiae were found in ticks, mites (Acari), fleas, and lice (Insecta). Furthermore, some rare strains of the four groups (SFGI, SFGII, CG and BG), as well as all those belonging to TG rickettsiae were identified in vertebrates such as humans or rats.

### Pan-chromosome and pan-plasmidome

Using predicted genes from both chromosomes and plasmids, we separately inferred the *Rickettsia* pan-chromosome of 34 species (80 strains) and pan-plasmidome of 19 species (41 plasmids). The pan-chromosome of *Rickettsia* spp. included 4073 cRiGs (orthologous clusters of chromosomal rickettsial genes) clustered from 93,691 protein-coding genes (117,821 CDSs) (see an example of the pan-chromosome patterns across ten genomes in Supplementary Fig. S3), while the pan-plasmidome of *Rickettsia* spp. contained 457 pRiGs (orthologous clusters of plasmidic rickettsial genes) grouped from 1502 protein-coding genes (1893 CDSs).

Within the pan-chromosome, only 15% (599) of cRiGs represented the core chromosome (present in all strains), whereas the remaining 85% (3474) of cRiGs corresponded to the flexible chromosome (present in a subset of strains). In the pan-plasmidome, we did not find any pRiG depicting the core plasmidome (present in all plasmids) indicating that 100% (457) of pRiGs constituted the flexible plasmidome (present in a subset of plasmids). However, both the core and flexible chromosomes (or plasmidomes) exhibited proportions of the gene contents that were variably distributed across the five rickettsial phylogroups (SFGI, SFGII, TG, CG and BG) and their species. We found that between 31 and 53% of genes represented the core chromosome in SFGI group species (in which the smallest fractions were found in *R. buchneri* REIS and *R. hoogstraalii* RCCE3). These representations increased from 58 to 60% in CG species (*e.g.,* in *R. canadensis*) and to 70% in TG species (*e.g.,* in *R. prowazekii*) phylogroups. In BG species (*R. bellii*) core genes accounted for 43% of their gene contents.

**Figure 3:**
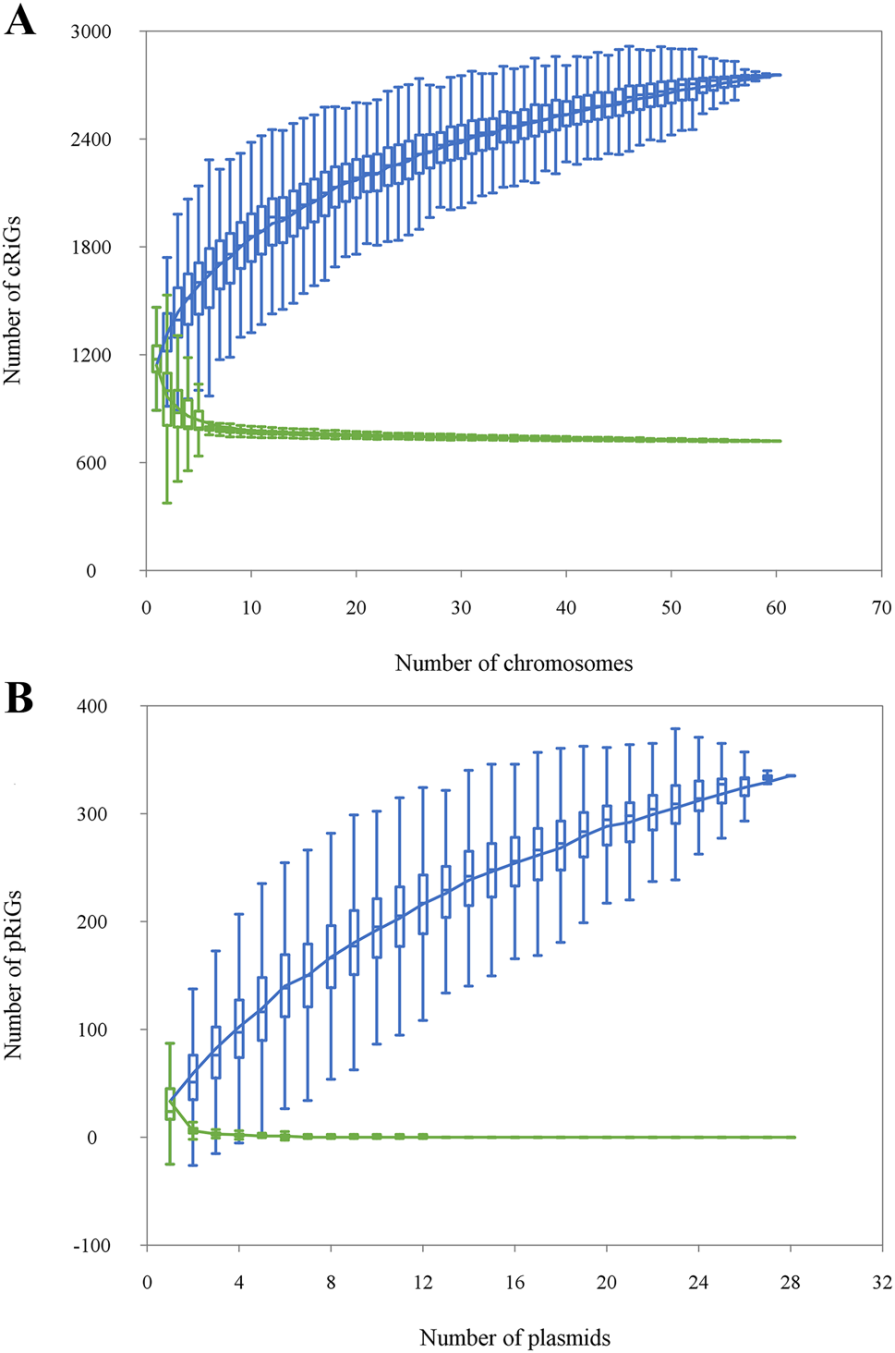
Estimation of the core chromosome (y = 497 e^−0.312x^ + 737; R^2^ = 0.95) and pan-chromosome (y = 1029 x^0.23^ + 87; R^2^ = 0.99) established through the analysis of 60 complete chromosomes of 28 *Rickettsia* spp. (**A**). Estimation of the core plasmidome (y = 144.2 e^−1.48x^ + 0.36; R^2^ = 0.99) and pan-plasmidome (y = 100.7 x^0.42^—74.3; R^2^ = 0.99) simulated from 27 complete plasmids representing 15 *Rickettsia* spp. (**B**). Box plots show the median and quartiles.

We found that the saturation curve of the core chromosome quickly became relatively constant, indicating that the addition of more chromosomes would not influence its stability (Fig. 3A). However, the saturation curve of the pan-chromosome gradually increased with the increase in the number of chromosomes, but it seemed to reach a plateau at a later stage, suggesting that the *Rickettsia* pan-chromosome was comprehensively open, and that genomes from several additional strains would need to be included for it to be completely closed. When increasing the number of plasmids (from one to 28), the core plasmidome remained equal to zero, confirming the absence of genes common to all plasmids (Fig. 3B). However, the saturation curve of the pan-plasmidome was far from reaching a plateau, indicating that the *Rickettsia* pan-plasmidome is also open.

### Genome size, degradation and expansion

In our study, we examined the *Rickettsia* genome degradation (i.e., increase of pseudogenes or split genes) and expansion (i.e., increase in numbers of gene duplications, plasmids and prophages) together with their genome sizes across the five phylogroups in the chromosomes and/or plasmids.

#### Chromosomes

*Rickettsia* spp. displayed consistent differences in chromosome sizes between and within the five phylogroups: SFGI (from 1.2 to 1.7 Mb) to the SFGII (from 1.2 to 2.3 Mb), CG (1.15) and BG (1.5 Mb) (Fig. 1, Table S1). The TG phylogroup still exhibited the smallest chromosomes in the genus (1.1 Mb).

When we examined the chromosome degradations in these phylogroups, overall we found that the proportions of pseudogenes were comparable between four phylogroups (Fig. 1, Table S1): SFGI (from 29 to 42%, except in *R. buchneri* REIS), SFGII (from 25 to 35%, except in *R. felis* LSU), CG (from 27 to 29%) and BG (from 22 to 26%). However, we found consistent variations in the proportions of pseudogenes more within the SFGI and SFGII than in the CG, BG and TG rickettsiae. The latter phylogroup exhibited the smallest proportions of pseudogenes (from 13 to 18%). Two exceptional strains, *R. buchneri* REIS and *R. felis* LSU, exhibited the largest fractions of pseudogenes (50–52%) in the genus, suggesting that each may have chromosomes which are highly riddled by stop codons which may result from a neutral/adaptive event and/or an incomplete sequence assembly of highly repetitive chromosomes (see below).

**Figure 4:**
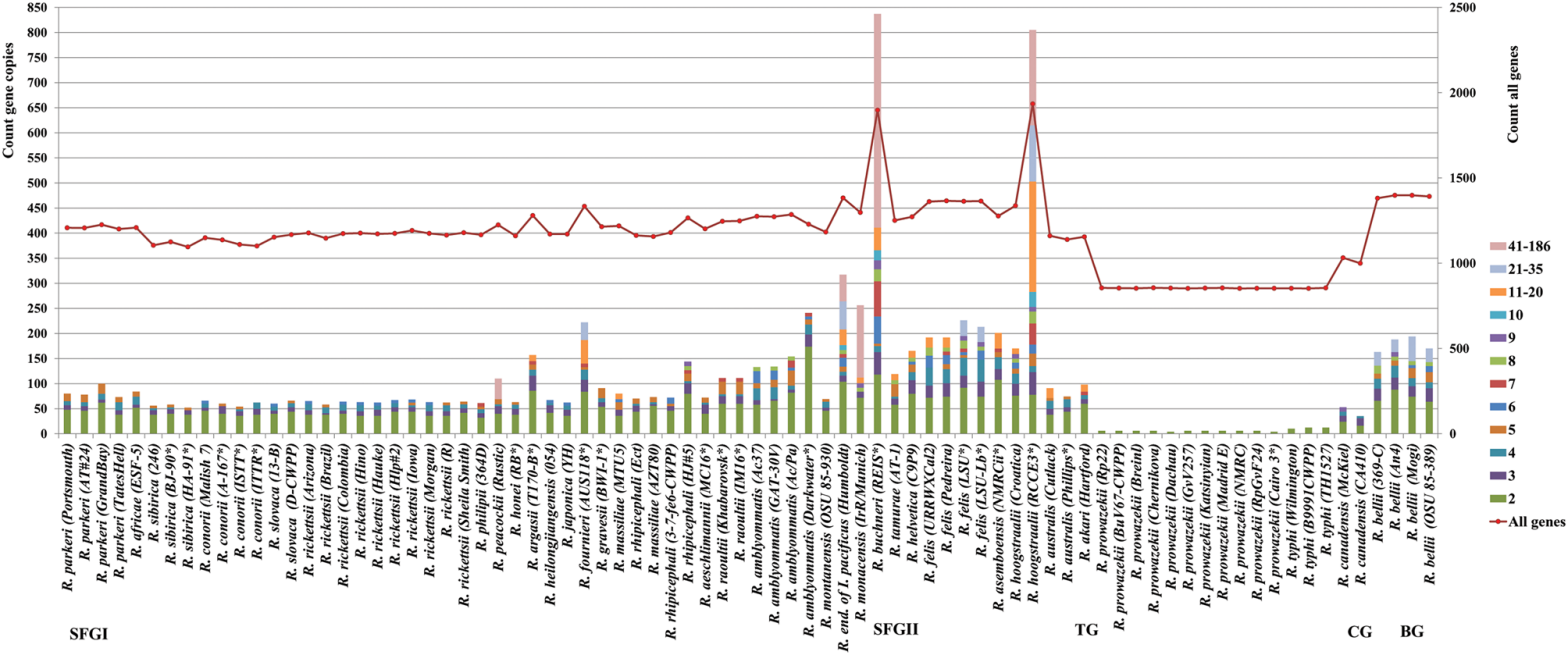
Distributions of gene proliferations identified in the pan-chromosome across 34 *Rickettsia* spp. (80 strains or chromosomes). The x-axis represents the 80 *Rickettsia* chromosomes examined. The left y-axis shows the total number of gene copies present in each chromosome. For each chromosome, colours indicate the numbers of genes according to their copy counts (from 2 to 186). The right y-axis displays the total gene count per chromosome.

Moreover, the analysis of the chromosome expansions across the five phylogroups revealed that the total numbers of chromosomal gene duplications were remarkably variable and higher in the SFGI (from 52 to 837) and SFGII (from 74 to 805) groups (Fig. 4). They decreased with low variations in the BG group (from 163 to 194) and much more in the CG (from 35 to 53) and TG (from 4 to 12) groups. Similarly, the frequency of chromosomal gene amplifications increased in the SFGI (from two to 186 copies) and SFGII (from 2 to 104 copies) groups and decreased in the BG group (from two to 27 copies) and much more in the CG (from two to nine copies) and TG (two copies) groups. Two strains of the SFGI and SFGII groups, *R. buchneri* REIS and *R. hoogstraalii* RCCE3 were a remarkable illustration of a massive genome expansion in the genus.

We then analysed the possible contribution of prophages in the chromosome sizes of some species representatives of each phylogroup that exhibited large variations in their chromosome sizes (Table S2). We detected no prophage in the smallest TG *R. prowazekii* Rp22 (1.1 Mbp) and CG *R. canadensis* (1.15 Mbp) chromosomes nor in the large SFGI *R. asembonensis* NMRCii chromosome (1.3 Mb). Moreover, all detected prophages were defective (either incomplete or questionable) and exhibited sizes ranging from 5.7 to 31.8 kb. When summing the sizes of these prophages for each species, we found that they represent less than 0.004% of the chromosome sizes in all phylogroups and species examined. As examples, the cumulated sizes of prophages represent only 0.0015%, 0.0004% and 0.0007% of the largest chromosomes, i.e*.*, *R. bellii* RML369-C (1.5 Mb, BG), *R. buchneri* REIS (1.7 Mb, SFGI) and *R. hoogstraalii* RCCE3 (2.3 Mb, SFGII), respectively.

#### Plasmids

Plasmid sizes also varied between and within the SFGI (from 12.3 to 121.4 kb), SFGII (from 19.4 to 63.8 kb) and BG (28.7 kb) phylogroups (Fig. 1, Table S1). The pRaf, pRae1 and pRma plasmids were the smallest plasmids in the *Rickettsia* genus (about 12–15 kb in sizes). Moreover, the numbers of plasmids were also variable and greater in the SFGI (from zero to four plasmids) and SFGII (from zero to three) groups compared to the BG group (zero to one).

Examination of the plasmid degradation revealed that the proportion of pseudogenes was highly variable in the SFGI (from 0 to 64.7%) and SFGII (from 17.9 to 58.6%) phylogroups (Fig. 1, Table S1). The largest proportions (> 50) of pseudogenes were found in several SFG plasmids, either complete or draft (*e.g.,* pRamA2, pReis4 and pRfeLs).

**Figure 5:**
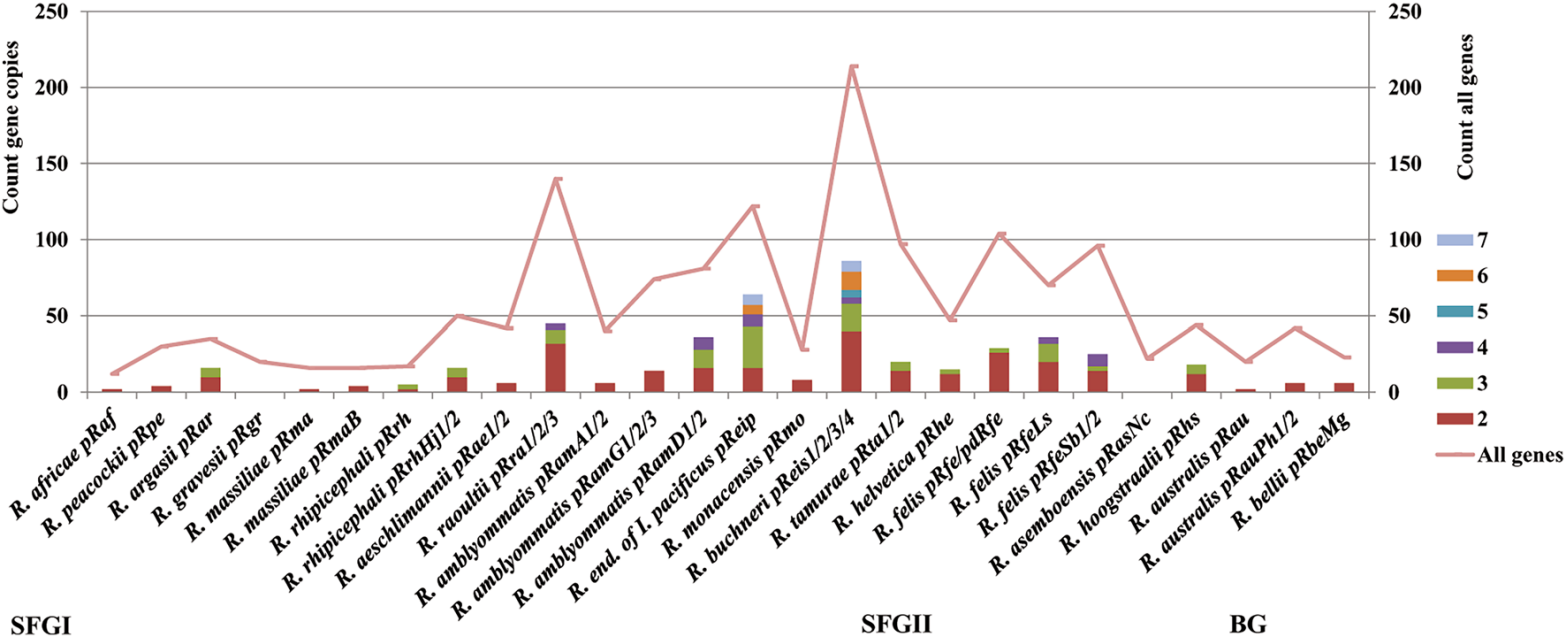
Distributions of gene proliferations identified in the pan-plasmidome across 19 *Rickettsia* spp. (26 strains, 41 plasmids). The x-axis represents the 26 *Rickettsia* strains, each harbouring from 1 to 4 plasmids. The left y-axis shows the total number of plasmidic gene copies present in each strain. For each plasmid, colours indicate the numbers of genes according to their copy counts (from 2 to 7). The right y-axis displays the total plasmidic gene count per strain.

The distribution of the total numbers of gene duplications in plasmids varied within the SFGI (from two to 64) and SFGII (from four to 36) phylogroups (Fig. 5). However, the frequency of gene proliferations in plasmids was low (from two to seven copies).

### Evolutionary networks

**Figure 6:**
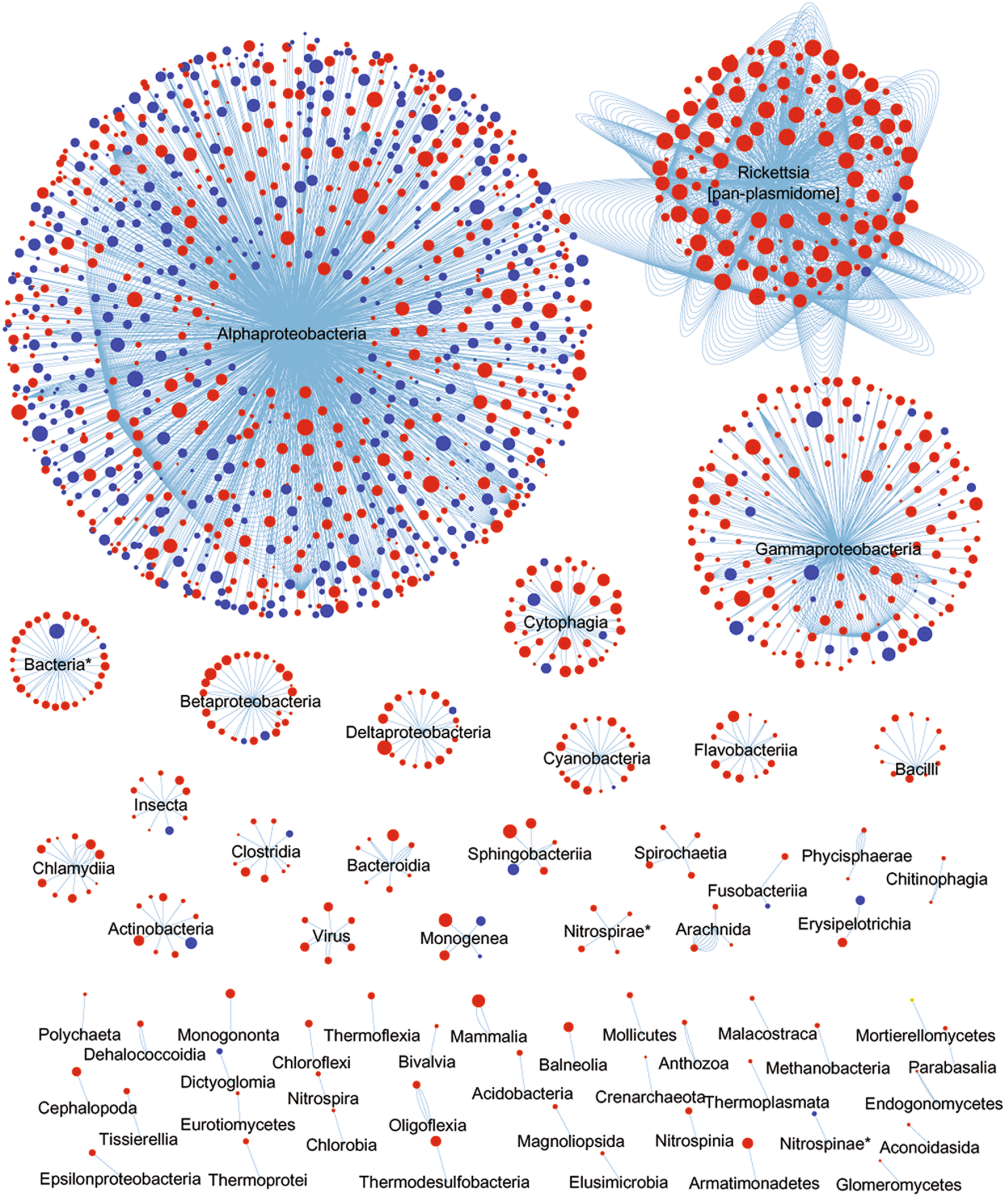
Evolutionary network of the *Rickettsia* pan-chromosome displaying best homologues from the *Rickettsia* pan-plasmidome and customised databases containing non-*Rickettsia* protein sequences of bacteria, archaea, eukarya and viruses. Nodes correspond to the pan-chromosome cRiGs. Node colours correspond to the core (in blue) or flexible (in red) cRiGs. Node sizes refer to sequence identities (from 30 to 100%) between a cRiG and its first best homologue, which are linked by blue edges. * unclassified bacteria.

**Figure 7:**
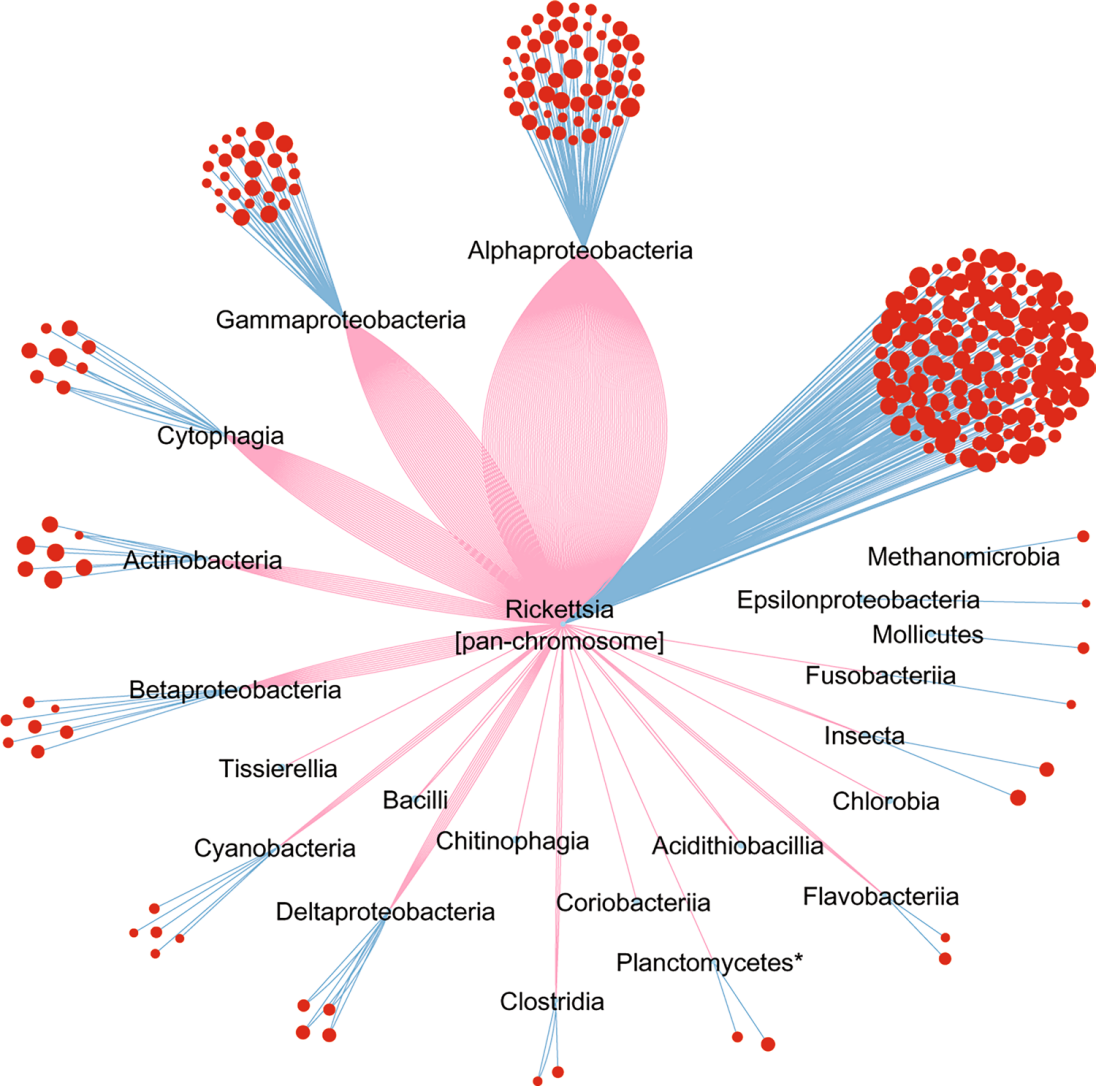
Evolutionary network of the *Rickettsia* pan-plasmidome exhibiting homologues from the *Rickettsia* pan-chromosome and customised databases containing non-*Rickettsia* protein sequences of bacteria, archaea, eukarya and viruses. Nodes represent the flexible pan-plasmidome pRiGs (in red). Node sizes refer to sequence identities (from 30 to 100%) between a pRiG and its first best homologue (blue edges). Some pRiGs also displayed second best homologues (red edges).

To infer the evolutionary relationships of the *Rickettsia* genus, we constructed an initial homology network of the pan-chromosome (4073 cRiGs, including 599 core and 3474 flexible genes) by identifying the first best hit of each cRiG in the pan-plasmidome and non-*Rickettsia* lineages (Fig. 6). This network excluded several plasmidic genes that did not have any sequence match with the pan-chromosome. Then, a network of the pan-plasmidome (457 flexible pRiGs) was constructed by detecting the first and second best hits of each pRiG in the pan-chromosome and non-*Rickettsia* lineages (Fig. 7). This latter analysis distinguished three gene sets: a first gene set in which the pan-plasmidome matched the pan-chromosome; a second gene set in which the pan-plasmidome matched other lineages; and a third gene set in which the pan-plasmidome matched both the pan-chromosome and other lineages.

#### The pan-chromosome network

We found that 75% of the pan-chromosome displayed best sequence homologies (identities = 30–100%) with the pan-plasmidome and diverse bacterial, archaeal, eukaryotic and viral taxa (56 classes/105 orders/162 families) as well as other unclassified organisms (Fig. 6). Within this taxonomic pattern, the pan-chromosome shared best sequence similarities (34.6%, including 503 core and 906 flexible genes) with the α-proteobacteria (Fig. 6, see examples of genes in Table S3). Remarkably, a second large fraction of the pan-chromosome (25.3% including only three core and 1026 flexible genes) exhibited best sequence similarity matches with the pan-plasmidome. Interestingly, a third gene set of the pan-chromosome contained small proportions of genes (from 7.1 to 1.2%; including 60 core and 551 flexible genes) that exhibited a best hit with distant bacterial lineages largely belonging to γ-proteobacteria, cytophagia, and β/δ-proteobacteria (Fig. 6, Table S3). Smaller fractions of genes (from 0.9 to 0.02%) best matched 54 more distant taxonomic classes (either identified or unidentified) such as cyanobacteria, chlamydiia and viruses (Fig. 6, Table S3). The remaining pan-chromosome (25%) had no homologue with the pan-plasmidome and non-*Rickettsia* lineages available in the databases.

#### The pan-plasmidome network

Overall, 72% of the pan-plasmidome genes exhibited best similarities (ids = 30–100%) with the pan-chromosome, diverse lineages (15 classes/35 orders/47 families) and/or other unclassified organisms (Fig. 7), whereas the remaining 28% had no homologue with our datasets and databases. Among this taxonomic profile, 10% (as first gene set of the 72%) of the pan-plasmidome displayed best matches with the pan-chromosome only, and 18% (as second gene set of the 72%) exhibited best hits with α-proteobacteria (9%), followed by γ-proteobacteria (3.9%), cytophagia (0.9%), actinobacteria (1.3%), δ-proteobacteria (1.3%), β-proteobacteria (1.1%), and cyanobacteria (0.4%), and then with much smaller proportions with four lineages (0.2%) (Fig. 7, Table S3). The remaining 44% (as third gene set of the 72%) of the pan-plasmidome showed best sequence similarities with the pan-chromosome and several lineages including α-proteobacteria (19.1%), γ-proteobacteria (8.3%), cytophagia (5.4%), β-proteobacteria (2.4%), actinobacteria (2.2%), δ-proteobacteria (1.5%), cyanobacteria (0.7%) and other lineages (from 0.4 to 0.2%) (Fig. 7, Table S3).

## Discussion

In this study, we examined the diversity, evolution and adaptation of arthropod-associated *Rickettsia* spp. using genomics, phylogenomics and pangenomics data analysis from 34 species (80 strains).

Overall, using phylogenomic analysis we found that the *Rickettsia* genus has diverged into five distinct major groups: two Spotted Fever groups (SFGI and SFGII), the Typhus group (TG), the Canadensis group (CG) and the Bellii group (BG). Our phylogenomic tree corroborates other rickettsial phylogenies estimated from subsets of species, strains and/or core genes/proteins^2,21,22,26,30–36^. However, although some of these phylogenies included a novel rickettsial clade (from *Adalia*)^2,30,36^ and four others (from *Hydra* spp., *Torix* spp., *Rhizobius* spp. and meloidae)^2,4–6,30^, they would not impact our robust five rickettsial phylogroups. Indeed, the *Adalia* clade was located between the CG and BG groups, while the other four clades were all external to the BG group. The rare specimens^2,4–6,30,36^ previously clustered in these new clades were not included in our study because no genomic sequence of these bacteria was available. Therefore, the BG phylogroup represents the outgroup of our data set, as it is the earliest divergent group among our taxa.

The 80 *Rickettsia* strains that we studied were isolated from diverse hosts worldwide, including Acari, Insecta and vertebrates. Examination of the diversity of arthropods of these bacterial species across the five rickettsial phylogroups revealed that the SFGI, CG and BG *Rickettsia* spp. were mainly associated with ticks (Acari). However, some species of these groups were also identified in mosquitoes, fleas and/or sheep keds (SFGI in Insecta)^37–39^, and in beetles, mosquitoes and/or flies (CG and BG in Insecta)^2,40^. The examined SFGII rickettsiae were detected in ticks, mites (Acari), fleas or lice (Insecta) and, in the case of *R. felis*, in ticks, mites (Acari) and mosquitoes (Insecta)^3,39–43^. Other *Rickettsia* spp. of our dataset were found to be associated with vertebrates^2^. For example, the TG species (*R. prowazekii* and *R. typhi*) were identified in humans and rats, respectively. However, these species are known to be associated with fleas and/or lice (Insecta)^3^, but were also detected in ticks^44,45^. On the other hand, our study found that 55% of *Rickettsia* strains were associated with ticks.

Previous data indicated that *Rickettsia* species are mainly associated with acarian and/or insect hosts, with a predominance in ticks. This entails two evolutionary scenarios that might have occurred for the *Rickettsia*-host association. First, an ancestor of *Rickettsia* spp. might have been associated with an acarian and/or an insect prior to its diversification. This suggests that *Rickettsia* spp. may have been maintained and have diverged inside diverse arthropod species through vertical transmissions (transovarial and trans-stadial). However, co-speciation between *Rickettsia* and arthropod species appears to be ruled out by the incongruence of phylogenetic trees^31,46^. Moreover, some *Rickettsia* spp. were found in association with various species of acarians and/or insects, suggesting that a strict co-speciation process is unlikely^2^. Episodic host changes between acarians, insects and/or vertebrates may have occurred by blood feeding or directly from infectious faeces of arthropods during the diversification of *Rickettsia* spp.^2,33,42,47^. Therefore, the large proportion of *Rickettsia* strains (*i.e.,* 55%) detected in ticks does not necessarily indicate that they are more abundant in ticks than in other arthropods but may be related to the fact that ticks are more sampled than insects for medical but not environmental purposes.

Alternatively, a rickettsial ancestor might have originally been associated with other eukaryotic hosts, as suggested by recent studies that identified two novel rickettsial clades within the genus *Rickettsia*: the torix clade associated with amoeba, leeches, amphipods and arthropods, and the hydra clade associated with protists and unidentified environmental hosts^2,4–6^. Both clades are ancestral outgroups to our five phylogroups, suggesting other possible host changes over time^2^.

Thus, all these data indicate that *Rickettsia*-host associations, diversity and ecology are complex and may reflect complex adaptations and trajectories. While arthropods are the most diverse animal phylum living in diverse ecological ecosystems^48,49^, using a limited diversity of both partners may seriously influence and undermine knowledge of the evolutionary history of *Rickettsia* and their hosts. Further studies on the evolution, diversity and ecology of *Rickettsia* and relatives, together with their partners, would improve our understanding not only of *Rickettsia*-host relationships but also of *Rickettsia* phylogeny and classification.

Regarding plasmids, which are mobile genetic elements harboured by prokaryotic cells, they replicate independently of the chromosome and act as a major driving force in prokaryotic diversity, evolution and fitness^50,51^. In this study, we identified 41 plasmids in members of the *Rickettsia* genus. The presence of these plasmids suggests two possible evolutionary scenarios regarding their origin. First, the ancestor of rickettsiae harboured one plasmid system (or more), that was(were) inherited and vertically transmitted to its progeny. Indeed, the 41 studied plasmids are present in three rickettsial phylogroups (SFGI, SFGII, and BG). In addition to these SFG species, *R. asiatica* and *R. akari* also harbour plasmids^52–54^, respectively. Moreover, although, it has been reported that there were no instances of plasmid-associated genes within TG rickettsial genomes^22^, our pangenomic study showed that the plasmidless TG *R. prowazekii* species (11 strains) harboured a gene cluster integrated in its chromosomes that best matched rickettsial plasmidic genes. Similarly, the SFG species *R. raoultii* (two strains, one with three plasmids and the other being plasmidless) exhibited a gene cluster that best matched a *R. peacockii* plasmid in its chromosomes. These results indicated that the TG *R. prowazekii* and the SFG *R. raoultii* species likely had one and a fourth plasmid, respectively, that were disrupted and integrated in their chromosomes during rickettsial evolution^28^. Overall, we bring evidence that rickettsial plasmids were present in four of the five rickettsial phylogroups as either individual replicons or plasmid genes integrated in the chromosomes. In addition, our supertree gathered plasmids into a single phylogenetic clade which is distinct from that of the chromosomes, suggesting that rickettsial plasmids originated from a common plasmid ancestor. Altogether, these results on 41 plasmids confirmed our previous work performed on 20 plasmids^28^. A potential ancestral plasmid was also suggested for the genus *Chlamydia*, another strictly intracellular pathogen of humans and animals^55^.

The alternative hypothesis that rickettsial plasmids may have been acquired laterally from other organisms^17,19–21,54^ does not appear to be relevant. Indeed, we identified several orthologous gene clusters that were common to most plasmids, and our supertree did not display any evidence of plasmid horizontal transfer, thus supporting only the vertical inheritance hypothesis^28^.

Rickettsial plasmids were identified in 56% and 33% of studied species and strains, respectively, at a rate of one to four plasmids. In contrast, 44% and 67% of studied species and strains had no plasmids, respectively. This result indicated that, during rickettsial diversification from ancestral lineages and a likely vertical transmission, plasmids were probably retained, multiplied and/or lost between ancestral and present lineages. The retention, multiplication and loss of plasmids may be due to evolutionary adaptations of rickettsiae to either single or several eukaryotic hosts during their obligate intracellular lifestyle. The rickettsial adaptation by the persistence and multiplication of plasmids (> = one replicon) in some strains suggests that most plasmids may harbour functionally indispensable genes (either laterally acquired and/or duplicated, see “Discussion” below) conferring upon them beneficial traits that could contribute to their survival and/or fitness in arthropod and/or vertebrate hosts. Moreover, the retention, multiplication and potential key roles of plasmids in several *Rickettsia* spp. make this genus remarkable in comparison with several closely related genera in the order Rickettsiales (families *Rickettsiaceae* [genera *Orientia, Occidentia, Phycorickettsia*], *Anaplamataceae* [*Anaplasma*, *Ehrlichia*, *Neorickettsia*] and *Candidatus* Midichloriaceae [*Candidatus* Midichloria, *Candidatus* Aquarickettsia]), which are devoid of plasmids^21,56–59^. Moreover, only a single putative plasmid was identified in the intracellular genus *Wolbachia,* another member of the family *Anaplamataceae*^60^. This plasmid is smaller (9.2 Kb) than rickettsial plasmids (> 12.3 kb) and does not match their most common gene, the DnaA-like gene. This suggests a possible distinct origin from that of rickettsial plasmids.

On the other hand, the rickettsial adaptation by the loss of plasmids in some strains may result from the fact that plasmids became non-essential and represented a biological cost for these bacteria. This adaptive loss of plasmids may be total and due to the reductive evolutionary process, which may confer to rickettsiae another strategy of survival and fitness inside their hosts. The adaptive loss of plasmids may also be partial by maintaining, after a reductive disruption, some gene clusters via lateral gene incorporation into the chromosomes, as shown for example, in the plasmidless *R. prowazekii*, and in the *R. raoultii* species^28^, as well as in a *Coxiella burnetti* strain^61^. The loss of plasmids may also be accidental or result from numerous passages of the bacteria in selective cell culture, as observed for *R. felis*^62^. Some species, such as *R. parkeri*, *R. typhi* and *R. canadensis*, which are represented by a small number of strains (< = four), all plasmidless, require exhaustive population genomic investigation to confirm their status, as has been carried out for *R. japonica*^63^ and *Orientia tustsugamushi,* the closest relative genus to *Rickettsia*^56^. Therefore, more genomic data are needed to improve our knowledge of the diversity and distribution of plasmids in *Rickettsia* spp.

Genome reduction (or shrinkage) and expansion are other evolutionary processes that shape bacterial evolution, adaptation and diversity, leading to either their simplification or complexification^11,19,64^. Genome reduction is known to have occurred during the transition of bacteria from a free-living to obligate intracellular lifestyle^11,64^, and can result from both degradation or pseudogenisation and the loss of non-essential genes (either neutral ratchet or adaptive) under relaxed selective pressure and genetic drift^65–70^. However, genome expansion may take place by gene duplication and amplification (GDA forming paralogues) and horizontal gene transfer (HGT forming xenologues)^71,72^. Several studies showed that the genus *Rickettsia* undergoes reductive evolution^10–14,19,20,28,29,31,34^, but genome degradation and expansions remained less investigated in both the chromosomes and plasmids.

This study revealed consistent variations in the genome degradation and expansion between and within the five rickettsial phylogroups. Indeed, the SFG rickettsiae, followed by the BG rickettsiae, displayed the largest genomes in the genus and a high and differential degree of degradation and/or expansion by duplication in both the chromosomes and plasmids and/or an increase in plasmid numbers. It has been suggested that most gene expansions arrive via HGT and not via duplication^71^. The genome expansion identified in the SFG and BG species originated more from gene duplications and proliferations of both orthologous and/or xenologous genes. In contrast, the TG and CG rickettsiae have the smallest genomes in the genus and exhibited the lowest degree of genome degradation and expansions. These rickettsiae have the most reduced genomes in the genus^10–14,19,20^, and the loss of plasmids in these phylogroups may have contributed to this evolutionary process. Overall, these results show that the genus *Rickettsia* clearly fits with a general biphasic model of genomic evolution^68^ including genomic reduction by pseudogenisation and loss of genes and/or plasmids^13,14,19,20,28,29,31,34^, as well as genomic expansion by gene amplification^73,74^ and/or plasmid multiplication^28^. The SFG and BG species promote expansion rather than contraction, whereas the TG and CG species undergo a reduction rather than amplification. This suggests that members of the genus *Rickettsia* may have followed two distinct adaptive trajectories during its evolutionary history. Similar genomic evolutions were also described in several intracellular bacteria that underwent a genomic expansion (*e.g., Orientia tsutsugamushi*^56,58^*, Wolbachia* strain wFol^75^, *Burkholderia mallei*^67^ and *Cardinium* strain^76^) and/or a genomic contraction (*e.g., Candidatus* Fokinia solitaria^77^, *Buchnera aphidicola*, *Mycobacteium leprae*^67,78^, and *Chlamydia* spp.^79^).

SFG and BG rickettsiae as well as several closer or distant bacteria seemingly defied the reductive force which is characteristic of several symbiotic and/or pathogenic bacteria living in bottleneck intracellular niches. Moreover, SFG rickettsiae showed the highest core genome conservation in the genus as observed in the phylogenomic tree. All these results suggest that SFG and BG rickettsiae may be less dependent upon host cell resources for their survival and be less pathogenic than TG and CG rickettsiae. It may also explain why virulence in SFG rickettsiae may be multifactorial^80^. However, although the increase in virulence was associated with strong reductive evolution in TG rickettsiae^29^, the high level of core gene mutations observed in this phylogroup suggests that adaptive substitutions may also contribute to the pathogenicity and deadly diseases caused by these bacteria^25^, which requires further investigation.

Although they are intracellular bacteria exhibiting small genomes (from about 1.1 to 2.3 Mb), *Rickettsia* spp. exhibited a comprehensively open pan-chromosome and a completely open pan-plasmidome. Moreover, both *Rickettsia* “pans” shared genes with each other (25.3% and 39%, respectively) and/or α-proteobacteria (34.6% and 15%, respectively) as well as with distantly related and unrelated lineages largely belonging to γ/β/δ-proteobacteria, cytophagia, actinobacteria cyanobacteria, chlamydia, viruses and/or unknown taxa. These results suggest that rickettsial genomes harbour an important genomic diversity, and like several free-living prokaryotes^81^, *Rickettsia* spp. exhibited several genes which may have been laterally acquired (and duplicated or not) between their chromosomes, plasmids, some close α-proteobacteria relatives and/or distant lineages. Moreover, *Rickettsia* spp. contained an important proportion of genes (25–28%) that have no significant non-*Rickettsia* homologue in databases, suggesting the occurrence of more potential xenologues, whose origins remain to be discovered. More computational and experimental investigations are needed to determine the functions of critical genes for rickettsial survival and/or pathogenicity.

In addition, it is likely that arthropod-associated *Rickettsia* spp. have exchanged genetic material with microbiome members of these arthropods or other reservoirs in order to acquire genetic advantages and/or to amplify existing or new functions profitable for their survival, competition and/or fitness. Indeed, several lineages that shared homologous genes with *Rickettsia* spp. were previously identified among microbial communities in these ecological niches^82–87^. Among these taxa, *Wolbachia* (Rickettsiales), *Candidatus* Odyssella (Holosporales), *Maritalea* (Rhizobiales), *Francisella* spp., *Legionella massiliensis*, *Pseudomonas*, *Candidatus* Hamiltonella and/or *Berkiella* (γ-proteobacteria), *Chromobacterium* spp. (β-proteobacteria), *Lawsonia intracellularis* (δ-proteobacteria), *Cardinium* spp. (Cytophagia), *Spirulina major* (Cyanobacteria), *Candidatus* Protochlamydia amoebophila (Chlamydiia) and viruses (Caudovirales) can be cited as examples. Moreover, the mobile and integrative genetic elements found in rickettsial genomes, including 41 plasmids, 1036 transposase and integrase genes (23% of the pan-chromosome and 23% of the pan-plasmidome) and 178 conjugative *tra* genes (3.8% of the pan-chromosome and 5% of the pan-plasmidome)^19,36,58,73,74^, may have greatly orchestrated the strong cross-exchanges (or crosstalk), particularly for the SFG and BG phylogroups, and consequently the adaptation and fitness of *Rickettsia* spp. Thus, understanding the structure and dynamics of microbial communities in arthropods through metagenomic studies together with the mobile genetic elements will provide new insights into the dynamics of gene exchanges and interactions between *Rickettsia* and these micro-organisms.

## Conclusion

During their diversification and adaptation to eukaryotic hosts (mainly arthropods), rickettsial genomes may have been shaped by diverse evolutionary processes. This study is the first large comparative evolutionary genomic investigation of members of the genus *Rickettsia* carried out on fully re-annotated chromosomes (80) and plasmids (41) of 34 identified species. The study shows that rickettsial phylogroups, as well as species or strains, may follow common and/or specific adaptive trajectories for their survival inside arthropods and/or probably their pathogenicity for vertebrate hosts. *Rickettsia* evolved through genomic degradation and reduction, and/or genomic expansion punctuated by probably short, explosive and innovative episodes of complexification. This trade-off evolution in the genus *Rickettsia* corroborates the general model of contraction/expansion of organisms proposed by Wolf and Koonin^68^ and may be due to their long-term intracellular lifestyle and partnerships as well as to the probable switching of arthropod hosts and interaction with their microbiomes.

Using recently updated public databases, our current results provide a robust insight into the evolutionary relationships between the genus *Rickettsia* and diverse distant lineages, thus improving the results of similar previous studies performed on some rickettsial species such as *R. prowazekii*^13^*, R. bellii*^18^*, R. felis*^17,22,31,33,88^ and *R. massiliae*^89^*,* or groups of *Rickettsia* species^19,90^. Deeper investigations on population genomics, novel *Rickettsia* species and metagenomics of arthropods are needed to improve our knowledge of their diversity, ecology and evolution.

Our work has shed light on the complex genomic history of the obligate intracellular *Rickettsia* spp. It represents a comprehensive overview into post-genomic and experimental studies such as RNAseq and small regulatory RNAs^91–94^, proteomics^27,95–98^, genetic transformation^9^ and gene functions^16,99–101^ aiming at better understanding the molecular mechanisms of critical genes driven by evolutionary processes, which have influenced the adaptation, survival and virulence of *Rickettsia* spp.

## Material and methods

### Genome sequences and reannotation

A total of 34 *Rickettsia* species depicted by 80 strains were investigated in this study. Studied strains were isolated from diverse arthropod hosts, animals or humans (clinical patients) around the world (Table S1). Their publicly available genomic sequences were downloaded from the ftp server (ftp://ftp.ncbi.nih.gov/Genome/) of the National Center for Biotechnology Information (NCBI). Of all studied genomes, 61 were complete and 19 were draft sequences. To avoid potential biases across the originally data deposited in NCBI that were obtained by different gene prediction methods and annotation pipelines, we fully re-analysed the raw genomic sequences. First, we distinguished between unidentified contigs of plasmids and chromosomes. This was performed by aligning each sequenced draft genome against the plasmid contigs of the complete genomes using the MAUVE software^102,103^. Second, all separated chromosomes and plasmid contigs (27 previously and 14 newly identified plasmids) were subjected to a standard CDS (CoDing Sequence >  = 40aa) prediction with the AMIgene software^104^. Manual curation was then performed to reject false predicted CDSs. Automated functional re-annotation was performed against the RickBase^19^ and/or non-redundant protein databases using pipRick (an in-house annotation pipeline written in Perl language) according to the MicroScope Genome Annotation platform criteria^105^ and using the BLASTp algorithm^106^. Thus, two standardised databases from *Rickettsia* genomes and plasmids were constructed for further investigation. The prediction of the presence or absence of prophages in *Rickettsia* chromosomes was investigated using PHASTER (Phage Search Tool Enhanced release)^107^.

### Pangenome inference

To construct the *Rickettsia* pangenome, we separately inferred the pan-chromosome and pan-plasmidome in the 34 *Rickettsia* species (80 strains). For each “pan”, we first performed an all-against-all protein search using both OrthoFinder^108^ (with default parameters) and Proteinortho^109^ (Cutoffs: *E*-value = 10^–6^ and coverage ≥ 60%) software programmes. We then extracted consensus pan-chromosome and pan-plasmidome, each containing clusters of orthologues of chromosomal and plasmidic rickettsial genes (that we refer to here as cRiGs and pRiGs, respectively) but not ORFs or CDSs as follows:

(i) for both “pans”, we checked and uncollapsed each orthogroup (from OrthoFinder) with the help of cluster(s) of orthologous/paralogous genes (from Proteinortho) by performing visual/manual/scrutiny inspection and comparison of their accession numbers and/or protein sequence similarities and identifying clear cases of segregating paralogues which may be co-orthologues and not in-paralogues within a single orthogroup^30^. This strategy greatly helps the automatic and robust inference of pangenomic statistics of clusters of orthologous genes.
(ii) retrieve pseudogenes defined as either split genes or gene fragments (i.e., genes interrupted by frameshifts or internal stop codons with at least one CDS having a match coverage < 80%) using a reference protein having the longest aa sequences within each cRiG or pRiG, BLAST best matches and annotation^18,19,27,28^. Indeed, in highly degraded genomes, such as *Rickettsia* genomes, the recognition of orthology/paralogy for pseudogenes remains a difficult task for pangenomic prediction programmes^19,27^. They can mistakenly place orthologous/paralogous genes into distinct or single orthology groups, consequently leading to an overestimation of the statistics of orthologous/paralogous clusters and the gene contents of taxa in a pan-genome study.
(iii) discard some clusters displaying short (< 60 aa) and/or chimeric (< 100 aa) genes having no match or no significant hits with the GenBank database^19^.

Last, we estimated the pan-chromosome and pan-plasmidome sizes using the distance method implemented in the PanGP software^110^.

### Network analysis

To delineate networks of evolutionary relationships between the *Rickettsia* pan-chromosome or *Rickettsia* pan-plasmidome and microbial and non-microbial organisms, we first searched homologies between the pan-chromosome (4073 reference protein sequences of cRiGs) and the non-redundant protein database (excluding *Rickettsia* species) using BLASTp (*E*-value = 10^–6^, coverage >  = 60 and identity >  = 30%). The same approach was then performed for the pan-plasmidome using the same criteria. All best matches were visually examined. After that, the pan-chromosome was searched for homologies against a customised database (i.e., a database constructed from the best NR and plasmid homologues packaged into single protein FASTA files) using the same BLASTp criteria to identify the first and/or the second best homologues that could be either plasmids and/or other microbial or non-microbial genes. Finally, results of the last homology searches were summarised into evolutionary networks between the pan-chromosome, pan-plasmidome and microbial as well as non-microbial protein sequences using the CYTOSCAPE software^111^. A similar strategy was used for the construction of the evolutionary network of the pan-plasmidome (457 reference protein sequences of each pRiG) as compared to the pan-chromosome, as well as microbial and non-microbial protein sequences (excluding *Rickettsia* species) from the non-redundant protein databases using the same thresholds.

### Phylogenomic and supertree analysis

Multiple sequence alignment of the core chromosome (152,764 aligned sites of 461 cRiGs) was carried out using the MAFFT software^112^. The phylogenomic tree was obtained using the Maximum Likelihood method and the –m PROTGAMMAJTT parameter using raxmlHPC-PTHREADS, and then plotted with the iTOL^113^ and MEGA^114^ software programmes. Node robustness was estimated using Bootstrap (BP) analysis of 300 replicates.

To perform plasmid phylogenetic analysis and investigate their evolutionary origins, we first selected 5 genes common to several plasmids and identified using the pan-plasmidome analysis. Overall, we searched for complete genes that are present in plasmids. However, we removed genes that are degraded, as *Rickettsia* species evolved by reductive evolution through progressive gene degradation. Degraded genes or short gene fragments cannot be used in the construction of phylogenies as they can blur the signals. The list of the 5 plasmidic genes used in our analysis includes dnaA-like replication initiator protein, patatin-like phospholipase, helix-turn-helix DNA-binding domain, cell surface antigen Sca12 and heat shock protein. Then, we searched for the best homologous genes of these five genes using the Blast tool against our *Rickettsia* pan-chromosome gene and GenBank databases, as previously described^28^. Sequences of each of the 5 genes were subjected to multiple sequence alignment, and phylogenetic analyses using Neighbour-joining (NJ) or Maximum Likelihood (ML) methods. NJ and ML trees were performed using the JTT amino acid substitution matrix and the WAG model plus the Nearest-Neighbour-Interchange (NNI) under gamma (Γ) distribution, respectively. We then constructed a supertree from Neighbour-joining (NJ) or Maximum Likelihood (ML) newik trees of five selected genes using the Robinson-Foulds algorithm^115^. Supertree methods enable synthesizing collections of phylogenetic trees with incomplete taxon overlap into comprehensive trees, or supertrees, that combine the information contained in a set of input trees and include all taxa found in the input trees^115,116^.

## Supplementary Information

Supplementary Information 1.

Supplementary Information 2.

Supplementary Information 3.

Supplementary Information 4.
