# Supplementary material for: Genomic evolution and adaptation of arthropod-associated Rickettsia
Source: Sci Rep. 2022 Mar 9;12:3807. doi: 10.1038/s41598-022-07725-z (PMC8907221; doi:10.1038/s41598-022-07725-z)

## Supplementary Figures

## Genomic evolution and adaptation of arthropod-associated *Rickettsia*

Khalid El Karkouri, Eric Ghigo, Didier Raoult and Pierre-Edouard Fournier

**Supplementary Figure S1.** Examples of *R. helvetica* proteins displaying amino acid substitutions (A, trans-regulatory protein) and INDELS (B, ubiquinone biosynthesis O-methyltransferase) similar to TG, CG and/or BG *Rickettsia* spp., but distinct from SFG *Rickettsia* spp.

A

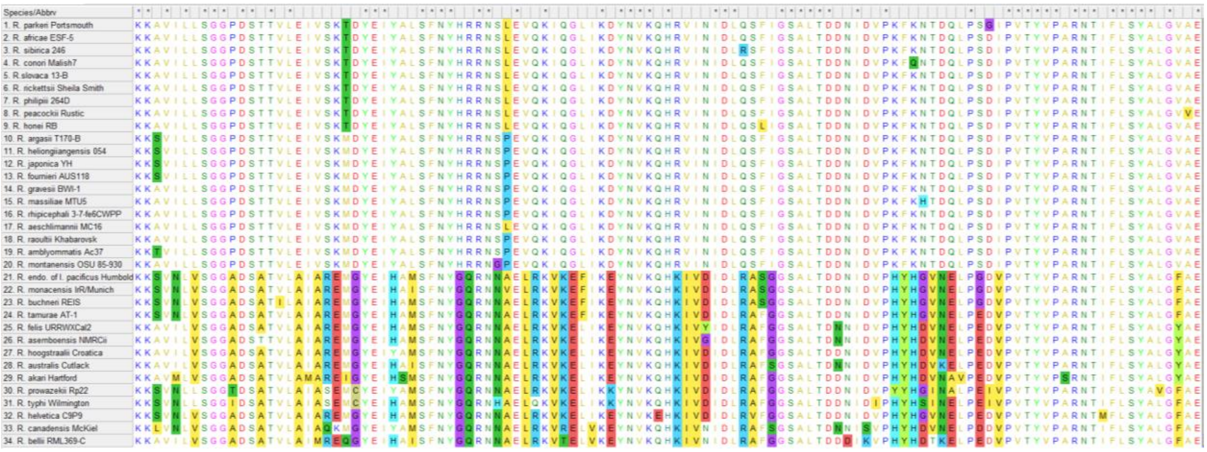

B

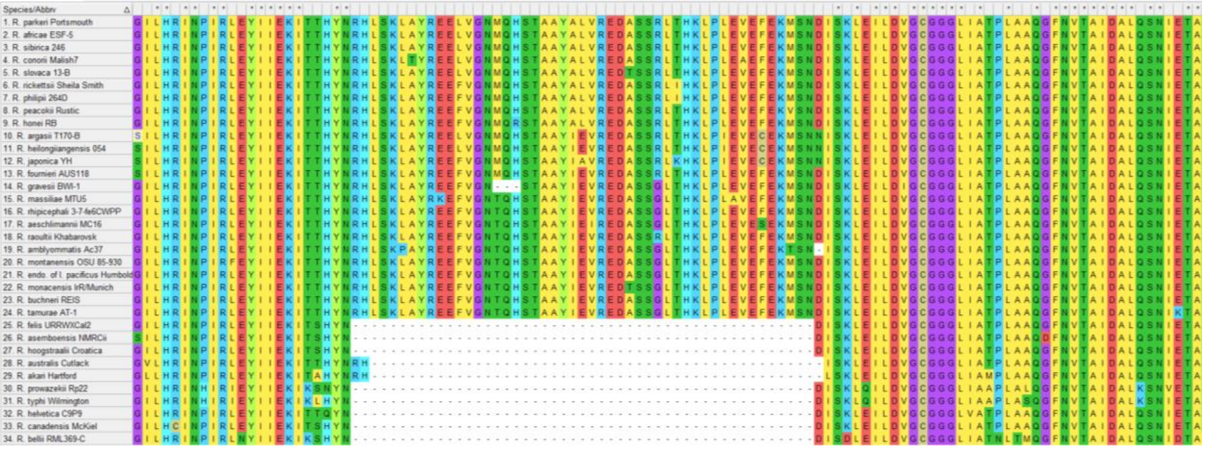

**Supplementary Figure S2.** Phylogenetic trees of *Rickettsia* plasmids and chromosomes inferred from five genes including DnaA-like replication initiator protein (A, 39 plasmids/26 strains and 17 chromosomes), patatin-like phospholipase (B, 13 plasmids/12 strains and 35 chromosomes), helix-turn-helix DNA-binding domain (C, 18 plasmids/13 strains), cell surface antigen Sca12 (D, 10 plasmids/10 strains and 30 chromosomes) and small heat shock protein (E, 21 plasmids/18 strains and 30 chromosomes). *Orientia tsutsugamushi* Ikeda (NC_010793.1), *Planococcus antarcticus* (ZP_10207867.1) and *Halobellus rufus* (WP_049986146.1) were used as best homologues to rickettsial genes. Bootstrap values that were lower than 60% were removed from the trees.

**A**

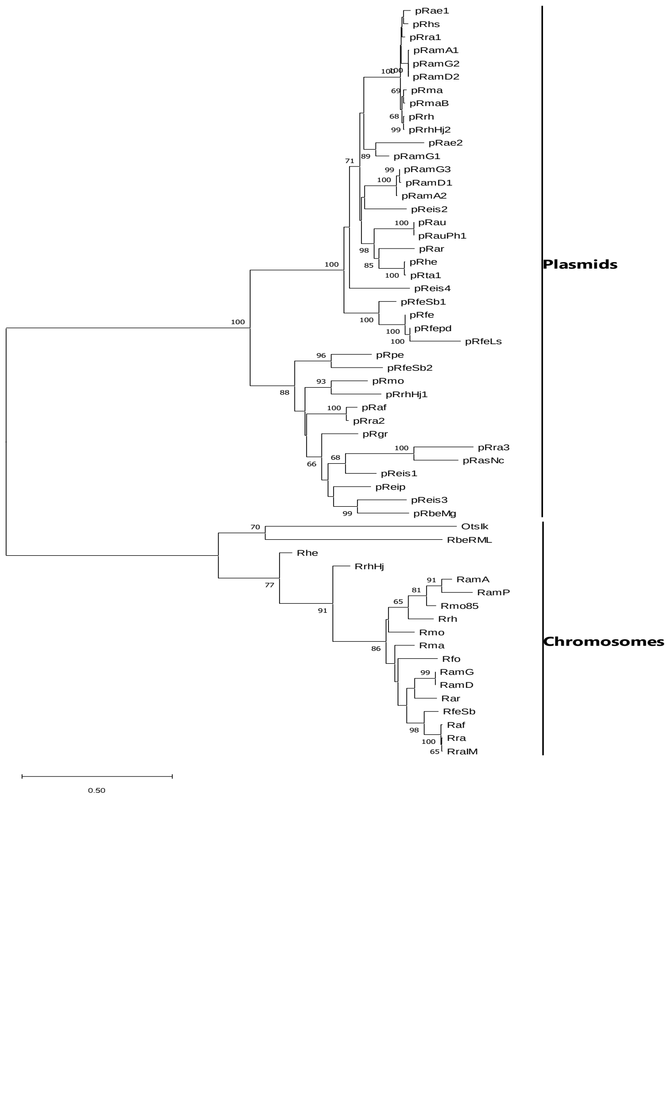

B

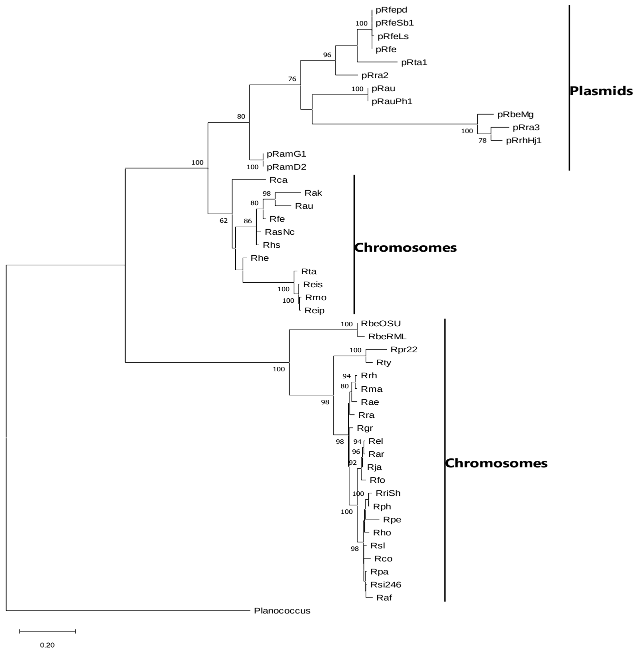
pRfepd
pRfeSb1
pRfeLs
pRfe
pRta1
pRra2
pRau
pRauPh1
pRbeMg
pRra3
pRrhHj1
Plasmids
pRamG1
pRamD2
Rca
Rak
Rau
Rfe
RasNc
Rhs
Rhe
Rta
Reis
Rmo
Reip
Chromosomes
RbeOSU
RbeRML
Rpr22
Rty
Rrh
Rma
Rae
Rra
Rgr
Rel
Rar
Rja
Rfo
RriSh
Rph
Rpe
Rho
Rsl
Rco
Rpa
Rsi246
Raf
Chromosomes
Planococcus
0.20

C

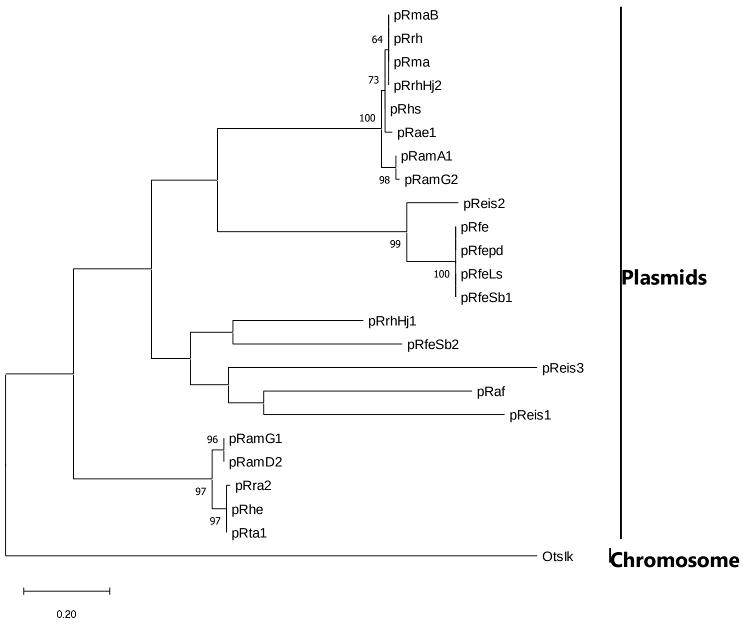
pRmaB
pRrh
pRma
pRrhHj2
pRhs
pRae1
pRamA1
pRamG2
pReis2
pRfe
pRfepd
pRfeLs
pRfeSb1
Plasmids
pRrhHj1
pRfeSb2
pReis3
pRaf
pReis1
pRamG1
pRamD2
pRra2
pRhe
pRta1
OtsIk
Chromosome
0.20

D

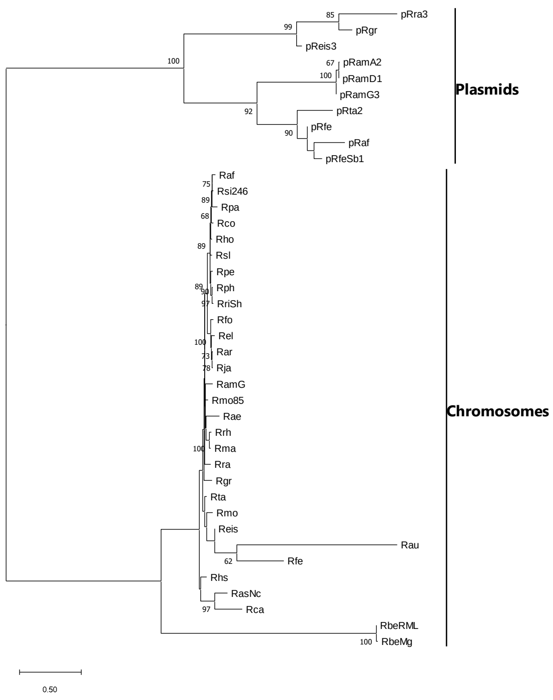
pRra3
pRgr
pReis3
pRamA2
pRamD1
pRamG3
pRta2
pRfe
pRaf
pRfeSb1
Plasmids
Raf
Rsi246
Rpa
Rco
Rho
Rsl
Rpe
Rph
RriSh
Rfo
Rel
Rar
Rja
RamG
Rmo85
Rae
Rrh
Rma
Rra
Rgr
Rta
Rmo
Reis
Rau
Rfe
Rhs
RasNc
Rca
RbeRML
RbeMg
Chromosomes
0.50

E

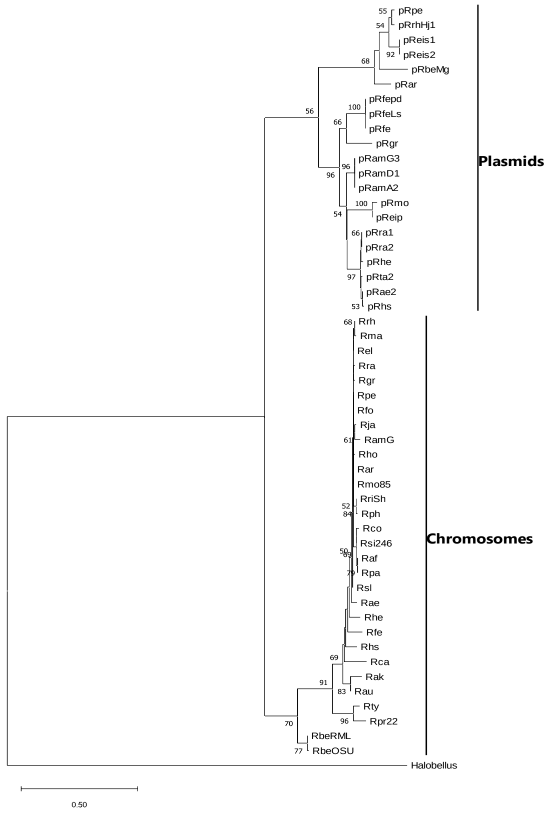
pRpe
pRrhHj1
pReis1
pReis2
pRbeMg
pRar
pRfepd
pRfeLs
pRfe
pRgr
pRamG3
pRamD1
pRamA2
pRmo
pReip
pRra1
pRra2
pRhe
pRta2
pRae2
pRhs
Plasmids
Rrh
Rma
Rel
Rra
Rgr
Rpe
Rfo
Rja
RamG
Rho
Rar
Rmo85
RriSh
Rph
Rco
Rsi246
Raf
Rpa
Rsl
Rae
Rhe
Rfe
Rhs
Rca
Rak
Rau
Rty
Rpr22
RbeRML
RbeOSU
Halobellus
Chromosomes
0.50

**Supplementary Figure S3.** Example of the pan-chromosome pattern obtained from complete chromosomes of 10 *Rickettsia* species belonging to the five major *Rickettsia* phylogenomic groups (SFGI, SFGII, TG, CG and BG). All species share 755 core genes and display a patchy distribution of flexible genes across them. Each blue column represents the total number of cRiGs common to at most 10 species.

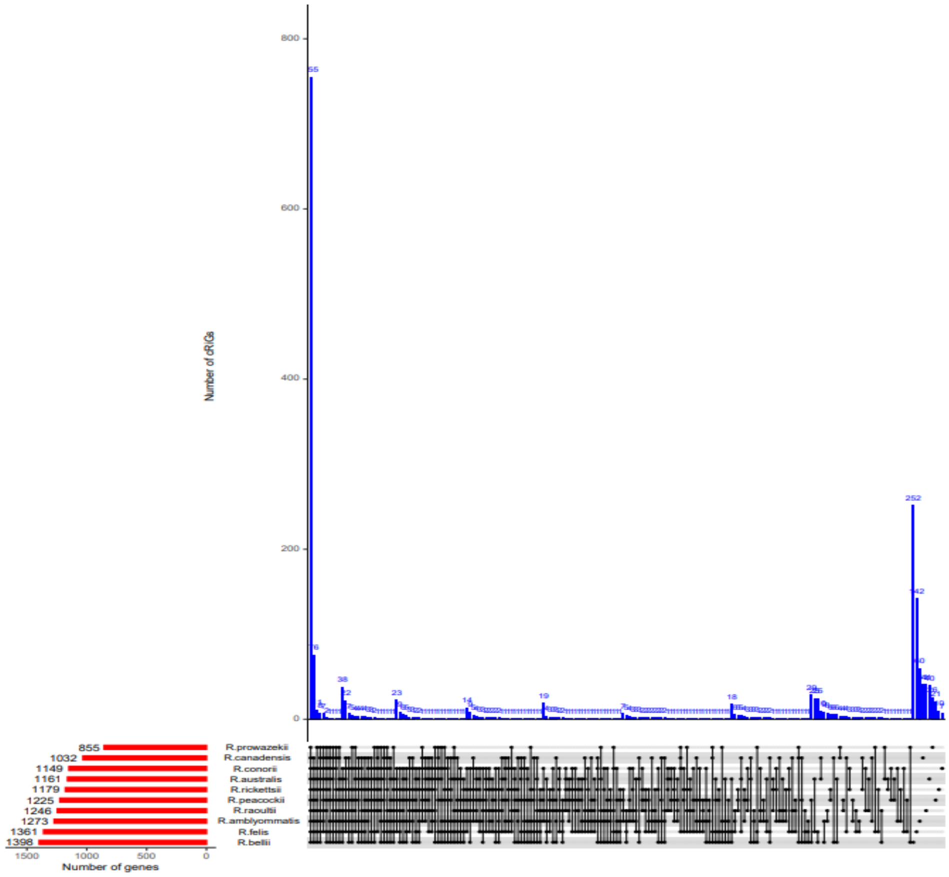

Supplement: Supplementary file 1 — Supplementary Information 1. [file 41598_2022_7725_MOESM1_ESM.pdf]
