# Supplementary material for: Genomic evolution and adaptation of arthropod-associated Rickettsia
Source: Sci Rep. 2022 Mar 9;12:3807. doi: 10.1038/s41598-022-07725-z (PMC8907221; doi:10.1038/s41598-022-07725-z)
Supplement: Supplementary file 2 — Supplementary Information 2. [file 41598_2022_7725_MOESM2_ESM.pdf]

Supplementary Table S1. General features obtained from the re-annotation of 80 *Rickettsia* genomes.

Species	Strain	Chromosome						Plasmid				
		**Name**	**Contig**	**Size (bp)**	**Gene**	**PsdG %**	**RNA**	**Name**	**Contig**	**Size (bp)**	**Gene**	**PsdG %**
R. africae	ESF-5	Raf	1	1,278,540	1209	33.4	40	pRaf	1	12,377	12	0.0
R. parkeri	Portsmouth	Rpa	1	1,300,386	1208	33.3	40	-	-	-	-	-
	AT#24	RpaA	1	1,300,534	1207	33.5	40	-	-	-	-	-
	GrandBay	RpaG	1	1,309,691	1226	33.7	41	-	-	-	-	-
	TatesHell	RpaT	1	1,300,383	1201	32.9	40	-	-	-	-	-
R. sibirica	246	Rsi246	1	1,250,021	1105	29.0	41	-	-	-	-	-
	BJ-90	Rsi90	12	1,254,013	1125	31.1	40	-	-	-	-	-
	HA-91	RsiMo	21	1,252,357	1096	33.2	40	-	-	-	-	-
R. slovaca	13-B	Rsl	1	1,275,089	1153	33.6	40	-	-	-	-	-
	D-CWPP	RslD	1	1,275,720	1168	31.9	40	-	-	-	-	-
R. conorii	Malish 7	Rco	1	1,268,755	1149	34.0	40	-	-	-	-	-
	A-167	RcoCa	25	1,260,331	1137	36.5	40	-	-	-	-	-
	ISTT	RcoIs	33	1,252,815	1110	38.3	39	-	-	-	-	-
	ITTR	RcoIn	10	1,249,482	1101	34.2	40	-	-	-	-	-
R. fournieri	AUS118	Rfo	10	1,447,739	1334	36.0	40	-	-	-	-	-
R. peacockii	Rustic	Rpe	1	1,288,492	1225	34.8	40	pRpe	1	26,406	30	30.0
R. philipii	364D	Rph	1	1,287,740	1167	32.2	40	-	-	-	-	-
R. rickettsii	Arizona	RriAr	1	1,267,197	1178	33.4	41	-	-	-	-	-
	Brazil	RriBr	1	1,255,681	1147	32.0	41	-	-	-	-	-
	Colombia	RriCo	1	1,270,083	1174	32.9	41	-	-	-	-	-
	Hino	RriHi	1	1,269,837	1177	33.0	41	-	-	-	-	-
	Hauke	RriHk	1	1,269,774	1172	33.0	41	-	-	-	-	-
	Hlp#2	RriHp	1	1,270,751	1175	33.0	41	-	-	-	-	-
	Iowa	RriIo	1	1,268,201	1192	33.9	41	-	-	-	-	-
	Morgan	RriMg	1	1,269,809	1175	33.2	42	-	-	-	-	-
	R	RriRr	1	1,257,005	1165	33.1	41	-	-	-	-	-
	Sheila Smith	RriSh	1	1,257,710	1179	33.3	41	-	-	-	-	-
R. honei	RB	Rho	11	1,268,758	1161	32.9	40	-	-	-	-	-
R. heilongjiangensis	O54	Rel	1	1,278,471	1171	32.8	40	-	-	-	-	-
R. argasii	T170-B	Rar	25	1,408,359	1280	38.5	40	pRar†	5	29,516	35	48.6
R. japonica	YH	Rja	1	1,283,087	1171	32.7	40	-	-	-	-	-
R. gravesii	BWI-1	Rgr	28	1,327,625	1214	35.2	40	pRgr†	1	19,874	20	10.0

R. raoultii	Khabarovsk	Rra	1*	1,344,642	1246	33.0	40	pRra1	1	20,840	24	45.8
								pRra2	1	83,219	86	34.9
								pRra3	1	34,583	30	20.0
	IM16	RraIM	1*	1,344,557	1248	32.8	40	-	-	-	-	-
R. aeschlimannii	MC16	Rae	14	1,270,362	1202	36.1	40	pRae1[†]	1	14,948	16	37.5
								pRae2[†]	1	26,886	26	38.5
R. rhipicephali	3-7-fe6-CWPP	Rrh	1	1,290,368	1180	34.3	40	pRrh	1	15,099	17	41.2
	Ect	RrhEc	1	1,266,919	1163	33.4	39	-	-	-	-	-
	HJ#5	RrhHj	1	1,406,075	1266	32.8	40	pRrhHj1	1	28,907	33	21.2
								pRrhHj2	1	13,650	17	47.1
R. massiliae	MTU5	Rma	1	1,360,898	1219	32.1	40	pRma	1	15,286	16	43.8
	AZT80	RmaB	1	1,263,719	1157	33.7	39	pRmaB	1	15,000	16	43.8
R. amblyommatis	Ac37	RamA	1	1,415,369	1275	34.3	40	pRamA1	1	18,046	17	41.2
								pRamA2	1	22,798	23	52.2
	GAT-30V	RamG	1	1,407,796	1273	34.6	40	pRamG1	1	31,974	32	50.0
								pRamG2	1	18,263	17	47.1
								pRamG3	1	22,851	25	44.0
	Ac/Pa	RamP	1	1,439,831	1286	36.2	41	-	-	-	-	-
	Darkwater	RamD	66	1,353,996	1229	42.5	37	pRamD1[†]	1	22,272	24	54.2
								pRamD2[†]	1	62,106	57	50.9
R. montanensis	OSU 85-930	Rmo85	1	1,279,798	1183	30.8	40	-	-	-	-	-
R. tamurae	AT-1	Rta	25	1,357,921	1251	33.9	40	pRta1[†]	1	75,325	76	21.1
								pRta2[†]	1	19,970	21	52.4
R. buchneri	REIS	Reis	17[s]	1,776,098	1898	50.4	41	pReis1	1	55,147	52	26.9
								pReis2	1	66,811	69	37.7
								pReis3	1	49,883	42	16.7
								pReis4	1	33,951	51	64.7
R. monacensis	IrR/Munich	Rmo	1	1,353,450	1298	39.2	38	pRmo	1	23,486	28	46.4
R. end. of I. pacificus	Humboldt	Reip	1	1,482,156	1383	36.1	40	pReip	1	121,421	122	31.1
R. helvetica	C9P9	Rhe	1	1,369,927	1272	35.1	40	pRhe	1	47,188	47	34.0
R. akari	Hartford	Rak	1	1,231,060	1155	34.5	39	-	-	-	-	-
R. australis	Cutlack	Rau	1	1,296,670	1161	33.3	40	pRau	1	26,610	20	50.0
	Phillips	RauPh	45	1,274,508	1139	34.4	40	pRauPh1	1	26,608	20	30.0
								pRauPh2[†]	13	19,451	22	45.5
R. felis	URRWXCal2	Rfe	1	1,485,148	1361	25.7	40	pRfe	1	62,829	63	33.3
								pdRfe	1	39,263	41	39.0
	Pedreira	RfePd	1	1,485,193	1366	26.0	40	-	-	-	-	-
	LSU	RfeLs	21	1,483,152	1363	52.1	37	pRfeLs[†]	1	63,818	70	58.6
	LSU-Lb	RfeSb	34	1,467,522	1364	34.4	39	pRfeSb1[†]	8	59,280	57	38.6
								pRfeSb2[†]	1	52,299	39	17.9

R. asemboensis	NMRCii	RasNc	87	1,355,051	1277	31.2	41	pRasNc	1	21,692	22	45.5
R. hoogstraalii	Croatica	Rhs	1	1,444,049	1337	30.0	40	pRhs†	1	40,763	44	45.5
	RCCE3	RhsRc	49	2,303,093	1935	34.2	38	-	-	-	-	-
R. prowazekii	Rp22	Rpr22	1	1,111,612	855	13.7	40	-	-	-	-	-
	BuV67-CWPP	RprBc	1	1,111,445	851	13.4	40	-	-	-	-	-
	Breinl	RprBl	1	1,109,301	853	15.8	40	-	-	-	-	-
	Chernikova	RprCk	1	1,109,804	856	13.7	40	-	-	-	-	-
	Dachau	RprDh	1	1,109,051	854	15.1	40	-	-	-	-	-
	GvV257	RprGv	1	1,111,969	852	14.3	40	-	-	-	-	-
	Katsinyian	RprKs	1	1,111,454	854	13.5	40	-	-	-	-	-
	Madrid E	RprME	1	1,111,523	855	14.7	40	-	-	-	-	-
	NMRC Madrid E	RprNc	1	1,111,520	852	18.2	40	-	-	-	-	-
	RpGvF24	RprRf	1	1,112,101	853	13.6	40	-	-	-	-	-
	Cairo 3	RprCo	20	1,113,970	853	14.2	40	-	-	-	-	-
R. typhi	Wilmington	Rty	1	1,111,496	853	14.1	40	-	-	-	-	-
	B9991CWPP	RtyBW	1	1,112,957	852	13.6	40	-	-	-	-	-
	TH1527	RtyTH	1	1,112,372	855	13.8	40	-	-	-	-	-
R. canadensis	McKiel	Rca	1	1,159,772	1032	28.9	40	-	-	-	-	-
	CA410	Rca410	1	1,150,228	1000	27.5	40	-	-	-	-	-
R. bellii	RML369-C	RbeRML	1	1,522,076	1381	23.4	40	-	-	-	-	-
	RML An4	RbeAn4	1	1,543,880	1398	22.7	40	-	-	-	-	-
	RML Mogi	RbeMg	1	1,549,351	1398	23.8	40	pRbeMg†	1	28,731	23	43.5
	OSU 85-389	RbeOSU	1	1,528,980	1392	26.1	40	pRbeOSU	n-s	n-s	n-s	n-s

In parentheses, number of scaffold (s). PsdG, means pseudogenes. RNA values were collected from GenBank database. – strain without any plasmid.

*, one contig with a single gap. ^{S}, scaffold. † newly identified and named plasmid contig (s). n-s, not sequenced plasmid (Gillespie et al. 2007/2008).
