# Supplementary material for: Genomic evolution and adaptation of arthropod-associated Rickettsia
Source: Sci Rep. 2022 Mar 9;12:3807. doi: 10.1038/s41598-022-07725-z (PMC8907221; doi:10.1038/s41598-022-07725-z)
Supplement: Supplementary file 3 — Supplementary Information 3. [file 41598_2022_7725_MOESM3_ESM.pdf]

Supplementary Table S2. Prediction of prophages in *Rickettsia* chromosomes.

**Phylogroup**	**Species**	**Chromosome size bp**	**Number of prophages**	**Size of prophages Kb**	**Total size Kb (%)**
TG	*R. prowazekii* Rp22	1,111,612	-	-	-
CG	*R. canadensis* McKiel	1,159,772	-	-	-
SFGI	*R. raoultii* Khabarovsk	1,344,642	2	6.7 / 8.1	14.8 (0,0011)
SFGI	*R. tamurae* AT-1	1,357,921*	1	31.8	31.8 (0,0023)
SFGII	*R. asemboensis* NMRCii	1,355,051*	-	-	-
SFGI	*R. massiliae* MTU5	1,360,898	2	26.6 / 21.7	48.3 (0,0035)
SFGI	*R.* endo. of *I. pacificus* Humbolt	1,482,156	1	5.9	5.9 (0,0004)
SFGII	*R. felis* URRWXcal2	1,485,148	2	6.5 / 7.5	14 (0,0009)
BG	*R. bellii* RML369-C	1,522,076	3	8.7 / 7.8 / 6.6	23.1 (0,0015)
SFGI	*R. buchneri* REIS	1,776,098*	9	6 / 6.5 / 5.7 / 8.1 / 7.3 / 9.3 / 8.2 / 7 / 10.9	69 (0,0039)
SFGII	*R. hoogstraalii* RCCE3	2,303,093*	1	15.4	15.4 (0,0007)

*, draft chromosome.
