# Supplementary material for: Genomic evolution and adaptation of arthropod-associated Rickettsia
Source: Sci Rep. 2022 Mar 9;12:3807. doi: 10.1038/s41598-022-07725-z (PMC8907221; doi:10.1038/s41598-022-07725-z)
Supplement: Supplementary file 4 — Supplementary Information 4. [file 41598_2022_7725_MOESM4_ESM.pdf]

Table S3. Example of genes sharing best similarities between the pan-chromosome, pan-plasmidome and/or closely or distantly related and unrelated lineages.

Network	Lineage	Gene	Sequence identity
***Rickettsia* pan-chromosome**	**α-proteobacteria:**		
	Orientia sp., *Occidentia* spp., *Wolbachia* spp. *Ca. Arcanobacter*	Transposases (*tnp*), integrases (*int*), integrative conjugative elements (*tra*)	34% - 93%
	Wolbachia spp.	Biotin synthase *bio*B	75%
	Ca. Odyssella	Protein phosphatase	66%
	Thalassospira or *Maritalea*	Hypothetical proteins	51% - 73%
	Pelagibacteraceae bacteria	Sugar kinases, ribokinase family	33%
	***Rickettsia* pan-plasmidome**	Transposases and integrases	33% - 100%
		Integrative conjugative elements	44% - 81%
		Small heat shock proteins	98.5%
		peptidase M50	59.7%
		Guanosine polyphosphate pyrophosphohydrolase/ synthetase	46% - 55%
		Thymidylate kinase	61.8%
		MFS-type sugar transporter	40% - 97%
		Proline/betaine transporter	50% - 60%
		Patatin-like phospholipase	62.3%
	**Distantly related or unrelated lineages**		
	Several lineages	Transposases and integrases	35% - 80%,
	γ-proteobacteria: *Francisella* sp., *Caedibacter*	Proteins of unknown functions	53% - 74%
	Cytophagia: *Cardinium* sp.	Type I polyketide synthase	77%
	β-proteobacteria: *Chromobacterium* sp.	Phenylalanine-4-hydroxylase	55%
	δ-proteobacteria: *Ca. Magnetomorum* spp.	Archaeal ATPase family protein	62%
	Cyanobacteria: *Spirulina major*	Phage-related protein	62%
	Chlamydiia: *Ca. Protochla. amoebophila*	Cell division protein Fic	66%
	Chlamydiia: chlamydiales	Histidinol-phosphate aminotransferase family protein	60%
	Viruses, caudovirales	MFS-type sugar transporters	49% - 63%

***Rickettsia* pan-plasmidome**	***Rickettsia* pan-chromosome or other related/unrelated lineages**		
	α/γ-proteobacteria and actinobacteria: several species	Transposases and integrases	36% - 92%
	α-proteobacteria, rickettsiales: *Wolbachia* spp.	Cluster of seven genes including the phytanoyl-CoA dioxygenase family protein and L-allo-threonine aldolase	47% - 79%
	γ-proteobacteria: *Serratia marcescens*	Protein of unknown function	67%
	Cytophagia: *Cardinium* spp.	HlyD family type I secretion periplasmic adaptor	64%
	Actinobacteria: *Streptomyces* spp.	Tetratricopeptide repeat-containing protein	88.7%
	***Rickettsia* pan-chromosome and other closer/distant relatives**		
	Several lineages	Transposases and integrases	31% - 100%
	α-proteobacteria, rickettsiales: *Wolbachia* spp.<br>δ-proteobacteria: *Lawsonia intracellularis*	Clusters of biotin genes *bio*A/B/C/D	53% - 77%
	α-proteobacteria: *Orientia tsutsugamushi*	Cluster of conjugative elements	36% - 67%
	γ-proteobacteria: *Legionella massiliensis*	Cluster of three genes including tyrosine-type recombinase/integrase, sodium/solute symporter family protein, GNAT family N-acetyltransferase	71% - 100%
	γ-proteobacteria: *Pseudomonas*,<br>Ca. *Hamiltonella* and/or *Berkiella*	Cluster of five genes encoding acetyltransferase, aromatic ring-hydroxylating dioxygenase subunit alpha, short chain dehydrogenase/reductase family protein, 3-oxoacyl-(acyl-carrier-protein) synthase III, acyl carrier protein and ABC multidrug transporter, permease/ATP-binding protein	63% - 98%
